# Predictive Modeling of PROTAC Cell Permeability with Machine Learning

**DOI:** 10.1021/acsomega.2c07717

**Published:** 2023-02-01

**Authors:** Vasanthanathan Poongavanam, Florian Kölling, Anja Giese, Andreas H. Göller, Lutz Lehmann, Daniel Meibom, Jan Kihlberg

**Affiliations:** † Department of Chemistry-BMC, Box 576, Uppsala University, 75123Uppsala, Sweden; ‡ Computational Molecular Design, Bayer AG, 42096Wuppertal, Germany; § Drug Discovery Sciences, Bayer AG, 13342Berlin, Germany; ∥ Drug Discovery Sciences, Bayer AG, 42113Wuppertal, Germany

## Abstract

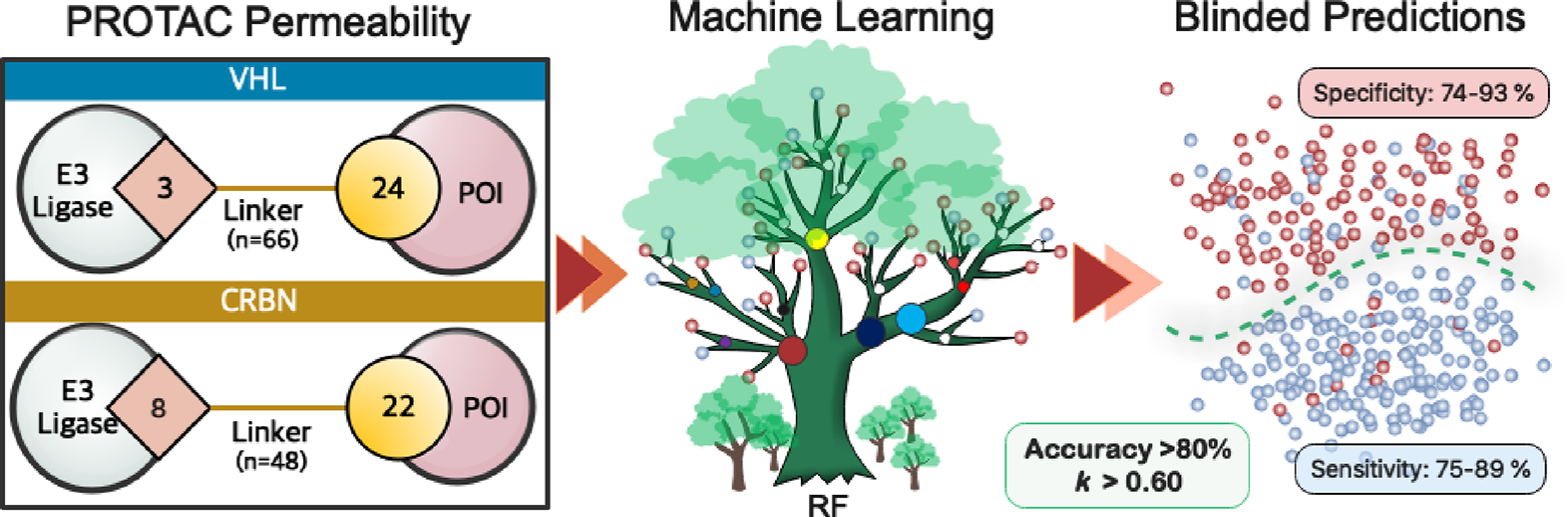

Approaches for predicting proteolysis targeting chimera (PROTAC) cell permeability are of major interest to reduce resource-demanding synthesis and testing of low-permeable PROTACs. We report a comprehensive investigation of the scope and limitations of machine learning-based binary classification models developed using 17 simple descriptors for large and structurally diverse sets of cereblon (CRBN) and von Hippel–Lindau (VHL) PROTACs. For the VHL PROTAC set, kappa nearest neighbor and random forest models performed best and predicted the permeability of a blinded test set with >80% accuracy (*k* ≥ 0.57). Models retrained by combining the original training and the blinded test set performed equally well for a second blinded VHL set. However, models for CRBN PROTACs were less successful, mainly due to the imbalanced nature of the CRBN datasets. All descriptors contributed to the models, but size and lipophilicity were the most important. We conclude that properly trained machine learning models can be integrated as effective filters in the PROTAC design process.

## 1 Introduction

Proteolysis targeting chimeras (PROTACs) consist of three domains: a ligand for the protein of interest (POI) and an E3 ubiquitin ligase ligand that are connected by a linker.^1^ PROTAC induced formation of a ternary complex with the POI and the E3 ligase results in ubiquitinylation of the POI and its subsequent degradation by the proteasome. The ability of PROTACs to modulate targets (POIs) considered as undruggable, e.g., due to the lack of well-defined binding sites, and to downregulate all the functions of the POI has stimulated an enormous interest in this new therapeutic modality. Most PROTACs reported in the literature contain either a von Hippel–Lindau (VHL) or a Cereblon (CRBN) E3 ligase ligand.^2^ However, all but one of the PROTACs that have entered clinical trials are based on CRBN.^1^

The mode of action of PROTACs requires that they permeate into target cells. Cell permeability is also required for PROTACs to be dosed orally, i.e., by the preferred route of administration of drugs. Achieving cell permeability and oral bioavailability for PROTACs is challenging as they reside in chemical spaces far beyond that defined by Lipinski’s rule^3^ of five and Veber’s^4^ rule,^5−7^ i.e., close to or outside the outer limits of the oral beyond rule of five (bRo5) chemical space.^8,9^ PROTACs based on a CRBN ligand populate the chemical space that overlaps with parts of the bRo5 space, while those based on VHL ligands are found even further from the oral drug-like space.^7^ For example, CRBN and VHL PROTACs have median MWs of approximately 900 and 1000 Da, respectively, with rotatable bond counts ranging from 20 to 30.^5,6^ Lipophilicities (cLogP) in a range of 4–6 and HBD counts of 3–4 are closer to those of orally absorbed drugs.

In view of the location of PROTACs in chemical space, approaches for prediction of their physicochemical and pharmacokinetic properties that can be applied as filters to reduce resource-demanding synthesis and testing should be useful in drug discovery projects. Predictive models for PROTACs are highly underdeveloped, but promising progress has been reported recently, albeit for small series of PROTACs. Thus, determination of the thermodynamic solubility of 21 PROTACs allowed the proposal of a random tree model for the classification of solubility based on the chromatographic lipophilicity and topological polar surface area (TPSA).^10^ The combined use of the parallel artificial membrane permeability assay and lipophilic permeability efficiency (LPE) provided insight into structure–permeability relationships for 11 PROTACs,^11^ while amide to ester substitutions consistently improved the permeability of a series of VHL PROTACs.^12^ A platform for PROTAC optimization was employed for the design and high-throughput synthesis of 91 CRBN PROTACs, the testing of which revealed that reduced numbers of HBAs and HBDs, in combination with cLogD values ≥4.0, increased cell permeabilities.^13^ Use of statistical methods for optimization of a series of CRBN-based Bruton’s tyrosine kinase (BTK) PROTACs suggested that reduction of the HBA count and of the flexibility of the linker increased the oral absorption.^14^

Information about conformational preferences has been found to improve predictions of cell permeability for moderately flexible compounds in bRo5 spaces such as macrocycles.^15,16^ Two recent NMR studies indicated that conformational preferences are also important for the permeability of PROTACs.^17,18^ However, PROTACs are often highly flexible and inclusion of conformational sampling in structure–property models may be challenging, as found in a recent investigation of PROTAC solubility.^10^ The modular construction of PROTACs from three units allows high-throughput synthesis of libraries with large structural diversity.^13^ Subsequent testing then generates large volumes of data, making approaches based on machine learning (ML) ideal for the derivation of structure–property models for PROTACs. In this work, we have investigated the use of ML for the construction of predictive binary classification models for PROTAC cell permeability. Our study relies on close to 700 VHL- and CRBN-based PROTACs having large structural diversity, for which cell permeabilities range from low to high. To the best of our knowledge, such comprehensive studies have not been reported previously. Therefore, we first describe our PROTAC datasets and then give in-depth accounts of how models were built and quality-controlled before evaluating their scope and limitations in the prediction of two blinded test sets. Moreover, we deduce some SAR for PROTAC cell permeability, even though we find it to be highly multifactorial, and finally discuss how the composition of the datasets have contributed to the predictability of the models. We hope that our work will stimulate further use of artificial intelligence in medicinal chemistry to unravel structure–property relationships that support the discovery of PROTACs entering into clinical studies.

## 2 Results and Discussion

### 2.1 Molecular Properties and Chemical Space of PROTACs

**Figure 1:**
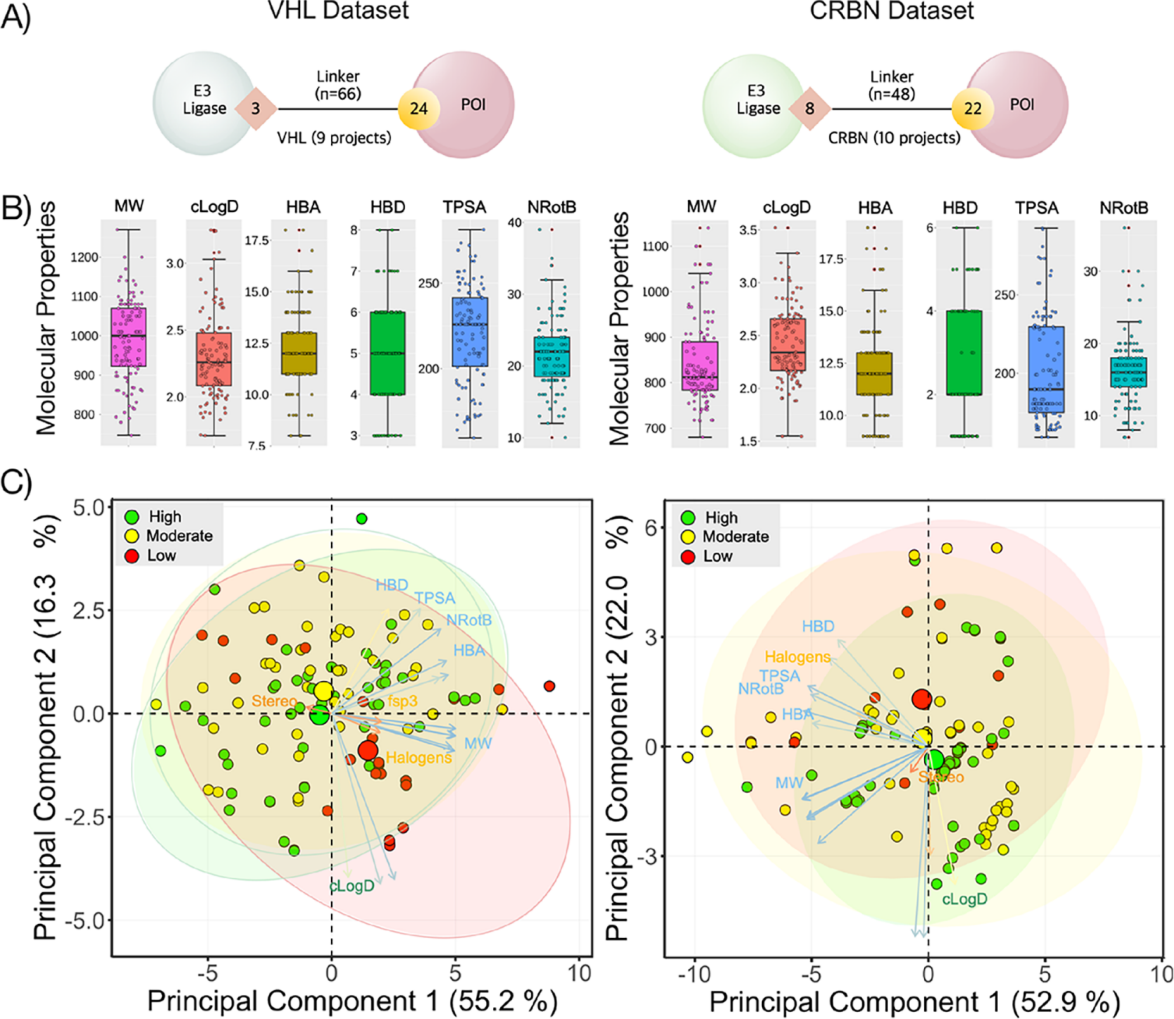
PROTAC datasets and their characterization. (A) Overview of the structural composition of the PROTACs in the VHL (*n* = 115) and CRBN (*n* = 113) sets. (B) Distribution of the molecular descriptors of Lipinski’s^3^ and Veber’s^4^ guidelines for the two sets. Box plots show the 50^th^ percentiles as horizontal bars, the 25^th^ and 75^th^ percentiles as boxes, and the 25^th^ percentile minus 1.5 × the interquartile range and the 75^th^ percentile plus 1.5 × the interquartile range as whiskers. Outliers are shown both as red dots and as circles in the color of the appropriate descriptor. (C) Score plots of the first two principal components from principal component analyses (PCAs), which describe 71.5% of the variance for the VHL set and 74.9% of the variance for CRBN. The PCAs were based on the 17 descriptors calculated for each PROTAC, which were subsequently used for construction of the permeability models (cf. Figure 3A). Ellipses in green, yellow, and red shading show the 95% confidence intervals for highly, moderately, and lowly permeable compounds, respectively. The centroid of each permeability class is indicated with a large circle in the color of the respective class. The contributions of individual descriptors to the PCAs are indicated by arrows.

A set of 228 PROTACs that showed a high degree of chemical diversity was used for development of cell permeability models. The PROTACs in the dataset were based on different VHL and CRBN E3 ligase ligands, a multitude of linkers, and a large number of POI ligands (Figure 1A). For example, the PROTACs in the VHL set have been assembled from three distinct E3 ubiquitin ligase ligands, 24 POI ligands, and 66 linkers. The cell permeability of each PROTAC had been determined as the ratio between the cellular and biochemical potencies for binding to the respective E3 ligase.^17,19^ This permeability surrogate is readily determined and used for selection of PROTACs for further evaluation but may be affected by intracellular binding to macromolecules and organelles. Based on the permeability ratio, each PROTAC was categorized into one of the following permeability classes, “high”, “moderate”, and “low” (Table 1). Considering the large structural diversity of the PROTAC datasets and that each PROTAC is annotated with cell permeability data generated under consistent conditions in one organization, these are high-quality datasets for construction of models for cell permeability using machine learning.

**Table 1:** Number of PROTACs Used for Data Analysis, Model Building, and Validation

permeability class	VHL^a^^,^^c^	CRBN^b^^,^^c^
high	46	59
moderate	44	45
low	25	9

a Cell permeability classification: <16 (high), 16–40 (moderate), and >40 (low).

b Cell permeability classification: <6 (high), 6–25 (moderate), and >25 (low).

c The rational for the permeability cut-offs are provided in Section 4.

In order to get an initial overview of the chemical space populated by the VHL (*n* = 115) and CRBN (*n* = 113) PROTACs, the distribution of the descriptors of Lipinski’s^3^ and Veber’s^4^ guidelines was calculated for the two datasets (Figure 1B and Figures S1 and S2). As expected from their large structural diversity, the compounds span an extensive descriptor space. For instance, the MW varied from below 800 to close to 1300 Da for the VHL set, while the number of rotatable bonds (NRotB) ranged from 10 to 39. Overall, the VHL dataset has a higher MW, contains more NRotBs and HBDs, and has a higher TPSA than the CRBN PROTACs. This is consistent with the fact that the VHL PROTACs in this study overall are less permeable (Table 1), and with that, VHL PROTACs in general reside further from the oral druggable space than CRBN PROTACs.^7^ We also performed PCAs to get additional insight into the chemical space populated by the two classes of PROTACs and how PROTACs differing in permeability are distributed in chemical space (Figure 1C). The PCAs were based on 17 descriptors calculated for each PROTAC, which were subsequently used for construction of permeability models (cf. Figure 3A below, Table S1). For both sets of PROTACs, there was no clear separation between the three permeability classes as data points were very scattered and to a large extent occupy the same chemical space. For this reason, developing multiclass classification models appeared very challenging. However, some observations from the PCAs suggest that it would be possible to construct binary classification models (BCMs), which would be valuable for rapid prioritization of compounds in drug discovery projects.

For the VHL dataset, the ellipsoids for the highly and moderately permeable PROTACs overlap, whereas the ellipsoid for the lowly permeable PROTACs is oriented differently (Figure 1C). Similarly, the centroids for the highly and moderately permeable compounds are located close to each other and far from the centroid of the lowly permeable group. As expected, the chemical space described by the descriptors of Lipinski’s^3^ and Veber’s^4^ guidelines overlapped for the three permeability classes (cf. Figures S1 and S2). However, there was a trend that lowly and highly permeable VHL PROTACs were distinguished by cLogD and TPSA (cf. Figures S1 and S2). Overall, this suggests that machine learning could succeed to construct classification models that distinguish between highly and lowly, or possibly between highly–moderately and lowly permeable VHL PROTACs. Characterization of the CRBN dataset using PCA and by chemical descriptors demonstrated that the molecular property space of the three permeability classes is very similar (Figure 1C and Figure S2), indicating that binary or multiclass classification modeling of this dataset could be complex.

**Figure 2:**
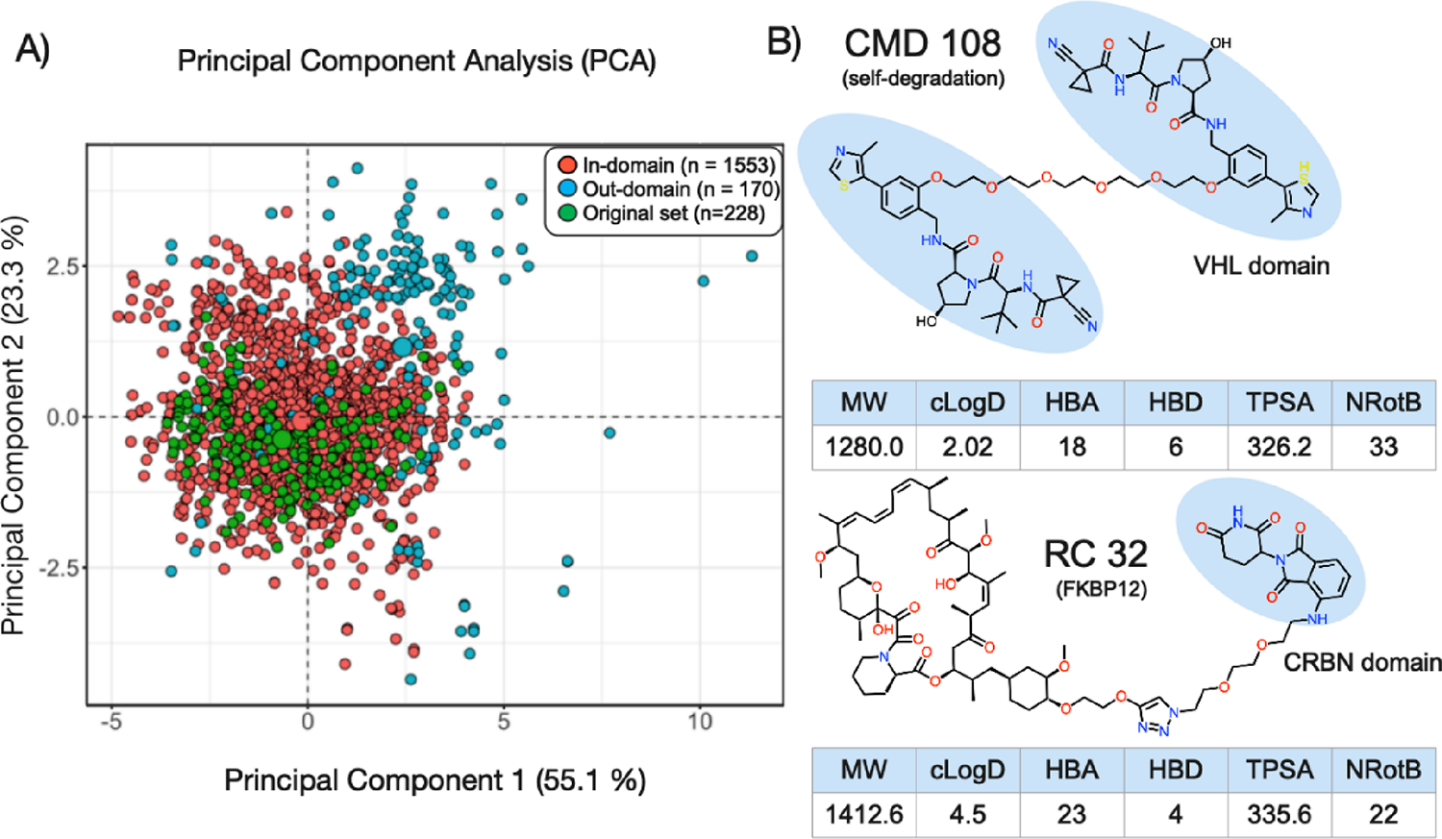
(A) Principal component analysis comparing the chemical space of PROTACs in the public domain (red and cyan circles) to our in-house set (green circles). Public PROTACs that are within the applicability domain of the in-house set are in red, while those outside are in cyan. The centroids for each set are indicated with a large circle in the color of the respective set. (B) Examples of molecular structures of two PROTACs that reside outside the chemical space of the in-house set. The descriptors of the Lipinski and Veber guidelines are given below the structure of each PROTAC.

The 228 PROTACs from this study and 1823 publicly available PROTACs^2,6^ were further analyzed to provide an understanding of the distribution of the two sets in the chemical space. The majority of publicly available PROTACs (>90%) were found to occupy the same chemical space (as described by the descriptors of Lipinski’s^3^ and Veber’s^4^ guidelines) as the current in-house dataset (Figure 2A, cf. Figure S3). In addition, the mean values for each descriptor were consistent across the in-house and public datasets. On the other hand, approximately 10% of compounds in the public set were located outside the chemical space defined by the in-house PROTAC set. The out-of-domain compounds contained macrocyclic PROTACs, and many of these PROTACs have complex POI ligands that for instance bind to the Bcl-XL or FKBP12 proteins (Figure 2B).

### 2.2 Construction of Classification Models

Artificial intelligence is being used to an increasing extent in drug discovery.^20^ As a result, some insight into how to make the best use of different methods throughout the complex process has emerged. Deep learning and neural networks require significantly larger datasets than those available in medicinal chemistry and may be complicated to set up. However, different ML methods have been found to be robust enough for building of ADMET classification models.^21,22^ It has also been reported that the quality of ML models improved only modestly when the training set grew.^21^ We therefore chose four state-of-the-art machine learning methods, i.e., decision tree (DT), kappa nearest neighbor (kNN), random forest (RF), and support vector machine (SVM), for the development of BCMs for PROTAC cell permeability.

**Figure 3:**
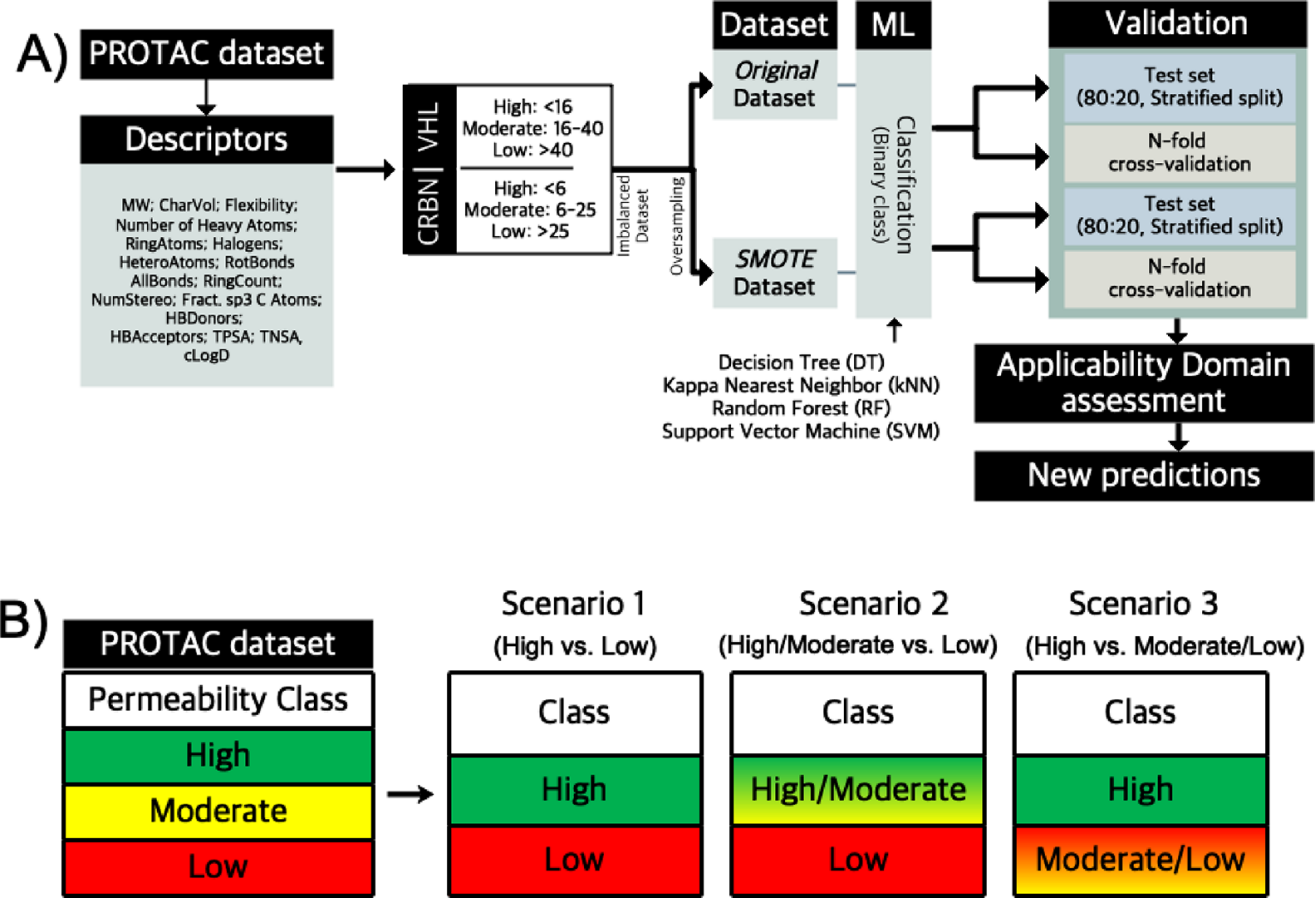
Methodology and associated datasets. (A) Overall workflow used for building classification models in the present study. The 17 descriptors are listed in the bottom left box, and the permeability cut-offs are in the box to the right of it. (B) Three different permeability class scenarios were used for the building of BCMs. Scenario 1 used only the highly and lowly permeable compounds, while in scenarios 2 and 3, the moderate class was merged with either the high or low class, respectively.

The KNIME open-source data integration platform, which is multifunctional and semi-customizable, was utilized for dataset preprocessing (normalization, filtering, merging, etc.), data analysis, and model construction (Figure 3A, Figure S4, and Table S2). “Global” cell permeability models were built from a dataset created by merging the VHL and CBRN datasets. The VHL and CRBN datasets were also used to construct individual classification models. Each model was constructed using a set of 17 molecular descriptors that were readily calculated from the SMILES structures of the PROTACs. The descriptors are well-established in medicinal chemistry and characterize the size, flexibility/rigidity, stereochemistry, and polarity/lipophilicity of the PROTACs (Figure 3A). Several of them are commonly used to assess the “drug-likeness” in the early phases of preclinical drug discovery projects, e.g., the descriptors of the rule of 5 and of Veber’s rule.^3,4^ Thus, if one or a few descriptors were found to be of high importance for the models, then this would provide insight useful in the design of cell permeable PROTACs. Additionally, classification models were built using an automatic attribute selection procedure (CfsSubsetEval, e.g., BestFirst) implemented in the Weka software.^23^ However, the resulting models did not provide any improvements and were not explored further (Table S3). Therefore, the models reported in this work are all based on the 17 molecular descriptors.

The CRBN dataset is highly imbalanced, i.e., the number of lowly permeable compounds is far lower than the number of moderately and highly permeable ones (Table 1). To a lesser extent, this is also the case for the VHL dataset. Imbalanced datasets may result in inaccurate or biased predictions when using conventional classification algorithms. In an attempt to avoid these issues, we used the SMOTE technique (synthesis of minority oversampling technique), which increases the number of data points in the minority class using the kappa nearest neighbor methodology, while preserving the majority class observations unchanged.^24^ BCMs were then constructed, both based on the original datasets and the SMOTE datasets, using the DT, kNN, RF, and SVM classifiers. The quality of the models was evaluated by their performance on “stratified sampling”-based internal test sets (25 random seedlings) and using 10-fold cross validation. In addition, each PROTAC dataset was employed for building BCMs in three scenarios differing by the presence or absence of the moderate permeability class in the training set (Figure 3B and Table S4). In the first scenario, the moderate permeability class was excluded from the dataset to investigate if this improved the predictability of the BCMs. Following that, the moderate-permeability class was merged with the high-permeability class (scenario 2) or the low-permeability class (scenario 3) to assess the impact on model quality.

The global cell permeability models, built by merging the VHL and CBRN datasets, did not appear promising based on the Cohen’s kappa value from the 25 random seedlings of the internal test set validations (Figure S5). Median kappa values ranged from 0.3 to 0.45 for the merged dataset, and the large distribution of the kappa values indicated that the models were unstable. Median kappa values improved to approximately 0.75 for permeability scenarios 1 (high vs low) and 2 (high/moderate vs low) but not for scenario 3 (high vs moderate/low) using the SMOTE approach. Instead, classification models for the individual VHL and CRBN datasets were investigated.

**Figure 4:**
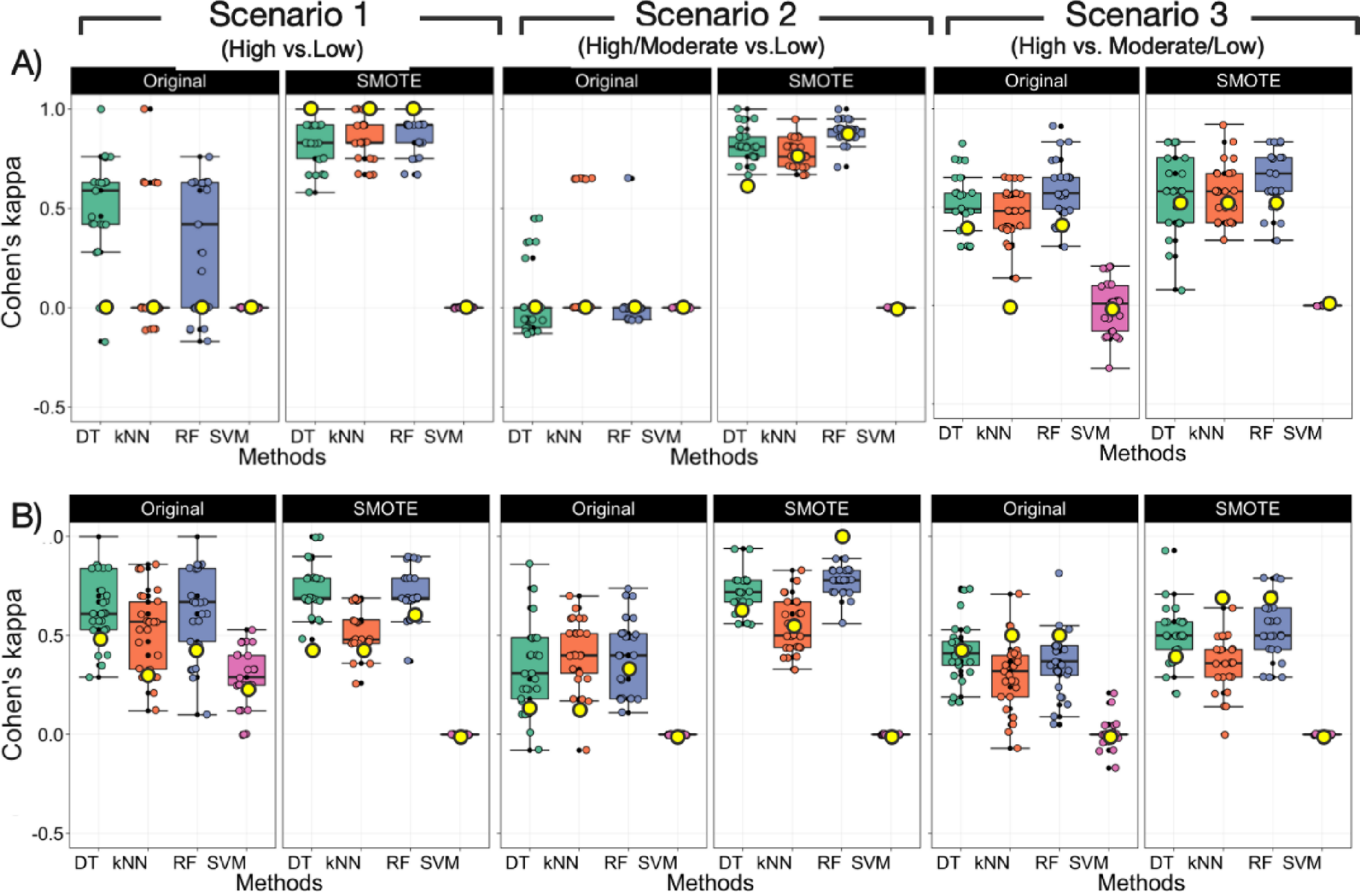
Cohen’s kappa statistics for internal test set validation of different BCMs for three permeability scenarios of (A) CRBN and (B) VHL PROTACs. Box plots show the kappa values from 25 random seedlings, while the yellow circles show the kappa values from 10-fold cross validation. In the box plots, the 50^th^ percentiles are marked as horizontal bars, the 25^th^ and 75^th^ percentiles as boxes, and the 25^th^ percentile minus 1.5 × the interquartile range and the 75^th^ percentile plus 1.5 × the interquartile range as whiskers. Outliers are shown both as black dots and as circles in the color of the method used to build the model. DT: decision tree, kNN: kappa nearest neighbor, RF: random forest, and SVM: support vector machine. Classification models can be assessed using the following cut-offs for Cohen’s kappa: κ < 0: no agreement, 0–0.19: poor agreement, 0.20–0.39: fair agreement, 0.40–0.59: moderate agreement, and 0.60–0.79 and 0.80–1.00: substantial to perfect agreement.^26^

For the CRBN dataset, DT and RF models for prediction of high vs low permeability (scenario 1) based on the original dataset appeared satisfactory based on the median value for Cohen’s kappa from the 25 random seedlings of the internal test set validations (κ, 0.59 and 0.42 for DT and RF, respectively; Figure 4A and Supporting Information Figure S5). However, the large distribution of the kappa values indicated that the models were unstable. Scenario 1 models constructed using the kNN and SVM methods were inferior (κ, 0 for both methods). With the exception of a few random seedling models, the models for prediction of high/moderate vs low permeability (scenario 2) were even worse. On the other hand, models for high vs moderate/low permeability (scenario 3) were interesting since all models with the exception of SVM yielded reasonable predictions. In particular, RF stood out with a median kappa value of 0.65 and a kappa range from 0.30 to 0.85 for the internal test set validations. The poor performance of the scenario 1, and in particular of the scenario 2 models, as compared to the scenario 3 ones can most likely be explained by the small size of the lowly permeable class (*n* = 9, cf. Table 1), which makes the scenario 1 and 2 datasets highly imbalanced. As might be expected, the quality of the scenario 1 and 2 models was significantly improved when the SMOTE approach was used to handle the imbalanced nature of these two datasets, whereas little improvement was obtained for scenario 3. Model assessment using 10-fold cross validation also clearly indicated that the predictability of BCMs built using the original dataset is the most uncertain for CRBN for scenarios 1 and 2. It should be noted that SVM models performed poorly both for the original and SMOTE datasets for all three scenarios.

Possible explanations for the poor performance of the CRBN models include the highly imbalanced nature of the dataset and the fact that the high-, moderate-, and low-permeability classes essentially occupy identical chemical space (Figure 1C). Both of these make the dataset extremely difficult for construction of BCMs. While the SMOTE approach may handle the dataset’s imbalance, oversampling techniques such as SMOTE can also result in overfitting due to class invasion.^25^ Thus, the SMOTE approach’s predictability can only be evaluated through the blinded prediction of additional test sets (cf. below).

For the VHL PROTACs, the quality of the models showed a large variation between the four methods and the three dataset scenarios as revealed by Cohen’s kappa (Figure 4B and Figure S6). Description of the model’s quality using the median value for Cohen’s kappa from the 25 random seedlings of the internal test set validations revealed that the DT, kNN, and RF methods provided satisfactory models (κ > 0.4) for prediction of high vs low permeability (scenario 1) when models were constructed using the original dataset. Little or no improvement was obtained with the SMOTE approach. However, the models obtained from the original dataset were characterized by more diverse kappa values between different seedlings of test set splitting (median κ of 0.57–0.67, with 0.43–0.72 at 90% confidence interval) than the models obtained from the SMOTE approach. Poor models were obtained from SVM, which originated from the fact that many lowly permeable compounds were misclassified as highly permeable ones. We have no explanation for this observation and have not succeeded in improving the SVM models, even after hyper-parameter tuning (data not shown). Assessment of the models using 10-fold cross validation led to similar conclusions as the kappa values fell within the 25^th^ and 75^th^ percentiles of the kappa range of the internal test set validation (Figure 4B).

BCMs were also constructed for the VHL dataset after merging the moderately permeable class with either the highly permeable class (scenario 2) or the lowly permeable class (scenario 3, Figure 4A). Models constructed using DT, RF, and kNN based on the original dataset were of somewhat lower quality for scenarios 2 and 3 (median κ = 0.3–0.4 for internal test set validations) than for the scenario 1 models. However, the quality of the models improved significantly with SMOTE for scenario 2 (median κ = 0.50–0.78) and to some extent also for scenario 3 (median κ = 0.36–0.50). Just as for the scenario 1 models, assessment of the scenario 2 and 3 models using 10-fold cross validation led to similar conclusions as for the internal test set validation. A possible explanation for the larger improvement with SMOTE for scenario 2 (high/moderate vs low) might be that the molecular property space of the moderate permeable class is closer to that of the highly permeable class as compared to the lowly permeable class (cf. Figure 1C). In addition, oversampling using SMOTE may result in overfitting as highlighted above for the CRBN models. Just as for CRBN and VHL scenario 1, models constructed using the SVM method were of poor quality and the SVM method was consequently omitted from the remainder of this study.

**Figure 5:**
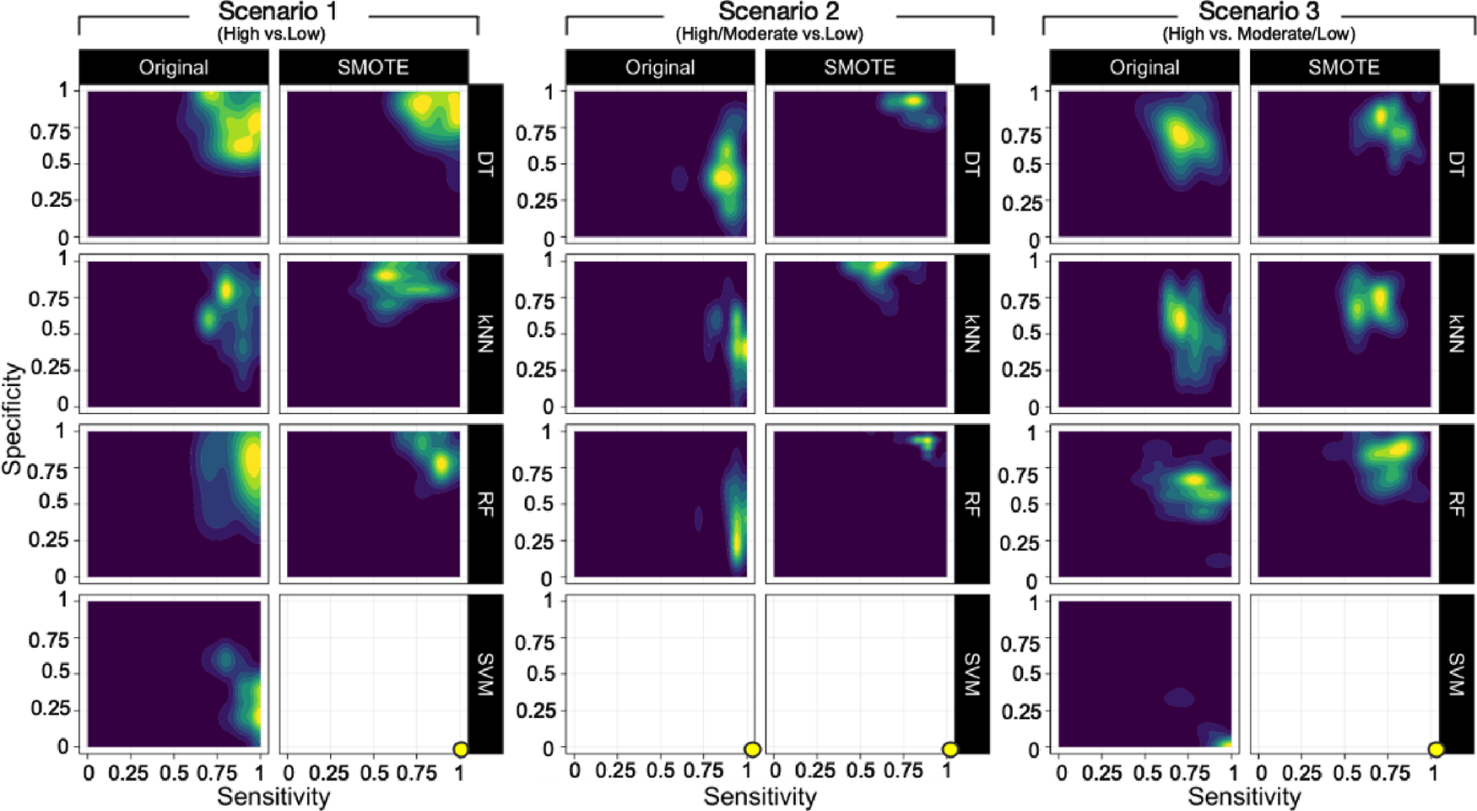
Sensitivity (correct prediction of high or high/moderate permeability) plotted against the specificity (correct prediction of low or moderate/low permeability) for the 25 random seedlings of the internal test set validation of the VHL models. Yellow indicates regions that are sampled with high frequency, while blue indicates those that are not sampled at all by the 25 random seedlings.

As described above, the quality of the permeability models for the VHL PROTACs showed a large variation between the four methods, whether SMOTE was used or not, and between the three permeability class scenarios (Figure 4B). Therefore, sensitivity (true positive rate or correct prediction of high or high/moderate permeability) was plotted against specificity (true negative class or correct prediction of low or moderate/low permeability) for each model to gain insight into the correct prediction and misclassification of the different BCMs (Figure 5).

Ideally, BCMs should be able to classify both classes correctly with sensitivity and specificity values close to 1.0. In scenario 1, in particular, the models obtained from DT and RF classified both highly and lowly permeable compounds correctly (Figure 5). In scenario 2, the models constructed using DT, kNN, and RF based on the original dataset showed good predictivity of the high/moderate class but not of the lowly permeable class. However, the SMOTE approach significantly improved the correct classification rate of the lowly permeable class. On the other hand, in scenario 3, the sensitivity and specificity of most models were moderate (∼50–75%), both when built using the original dataset and using the SMOTE approach. Interestingly, the consistently poor performance of the SVM models was found to originate from the failure to predict the low- and moderate/low- permeability PROTACs in all three scenarios.

**Figure 6:**
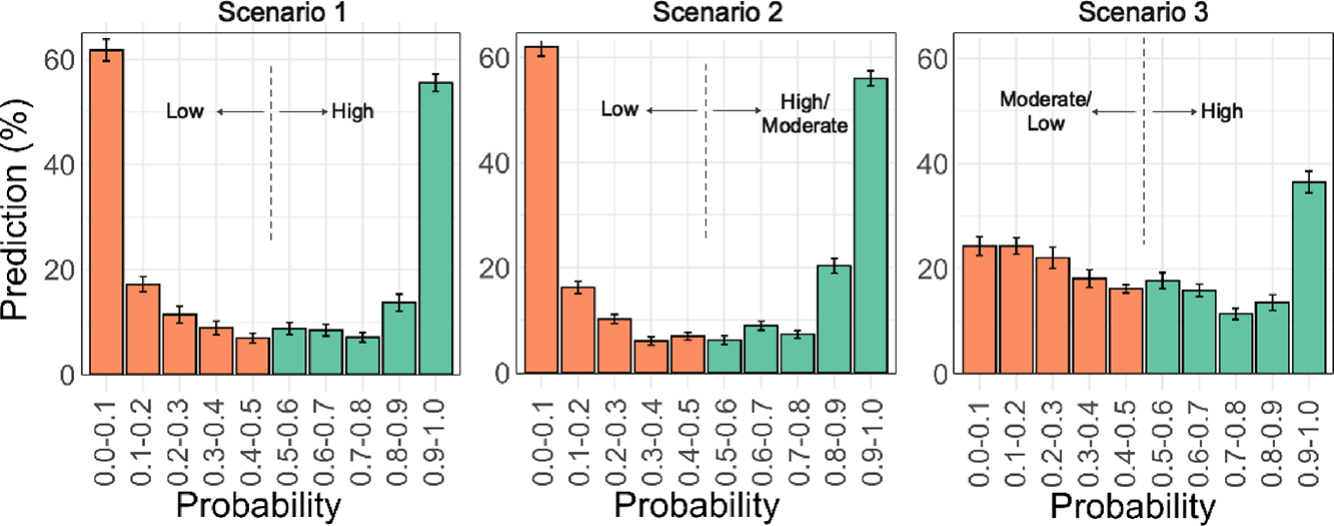
Probability distribution of true predictions for the random forest models built using the original VHL dataset. PROTACs having a probability smaller or larger than 0.5 were correctly classified as having low (orange) or high (green) permeability, respectively. A probability of 0.9–1.0 indicates that the compound was predicted to have a high permeability with >90% probability. Similarly, a probability of 0–0.1 indicates that the compound was predicted to have a low permeability with >90% probability.

Depending on the method used for construction of BCMs, the reliability of the models can be assessed using the probability distribution of true predictions.^23^ In brief, this means that each compound that has been correctly classified by the model is also annotated with the probability of belonging to that class. Due to their implementation in the KNIME platform, only models built using the RF classifier, but not with the DT, kNN, and SVM methods, provide probability distributions. The probability distributions of the scenario 1 and 2 RF models both peak at probabilities close to 0 and 1 (Figure 6), revealing these models to predict high versus low, or high/moderate versus low, permeability with excellent reliability. In contrast, the probabilities of correct predictions of high versus moderate/low permeability (scenario 3) are more evenly distributed, showing this model to be significantly less reliable than those for scenarios 1 and 2.

### 2.3 Prediction of Blinded PROTAC Sets

**Figure 7:**
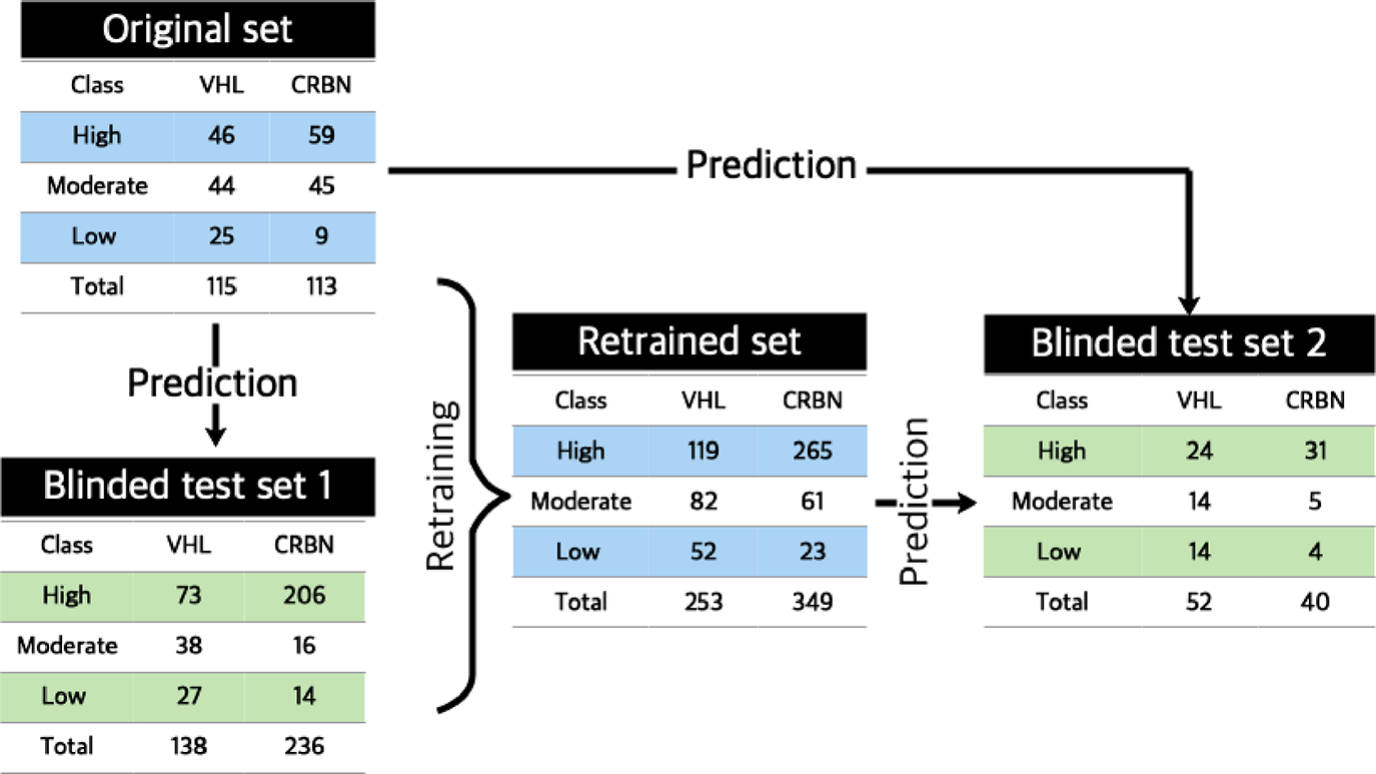
Number of compounds in the training sets of PROTACs used to construct BCMs (original and retrained set) and the datasets used as blinded test sets for validation of the models (blinded test sets 1 and 2). For each dataset, the distribution of compounds between VHL and CRBN PROTACs, as well as by permeability class, is given.

The applicability of the BCMs was assessed by predicting the permeability for two PROTAC test sets, for which the experimental permeability classification was kept blinded until after completion of the predictions (Figure 7). First, the applicability of the models built using the original dataset, and its SMOTE versions, was evaluated using a large set of VHL and CRBN PROTACs (blinded test set 1). Then, retrained models were built by combining the original and the first blinded test sets. The ability of the retrained models to predict the permeability of a second, smaller test set (blinded test set 2) was subsequently determined and compared to that of the original models.

Prior to predictions, the applicability domain (AD) of the original dataset for prediction of blinded test set 1 was evaluated (Figure S7). As a result, a diastereomeric pair of VHL PROTACs that resided outside of the AD was excluded from the test set to avoid uncertainty. Blinded predictions of the permeability of all of the remaining 138 VHL and 236 CRBN PROTACs (Table S5) were then performed using the models developed by the DT, kNN, and RF methods for the three permeability scenarios of the original training set (Figure 3B). Unblinding of the experimentally determined permeability data revealed that just over half of the VHL PROTACs in blinded test set 1 were highly permeable (Figure 7). The moderately and lowly permeable classes accounted for 27 and 19% of the VHL set, respectively. The CRBN blinded test set 1, on the other hand, was found to be highly imbalanced just as the original training set. The majority of compounds (>85%) were highly permeable, while the moderately and lowly permeable classes accounted for only 7 and 6% of the dataset, respectively.

**Figure 8:**
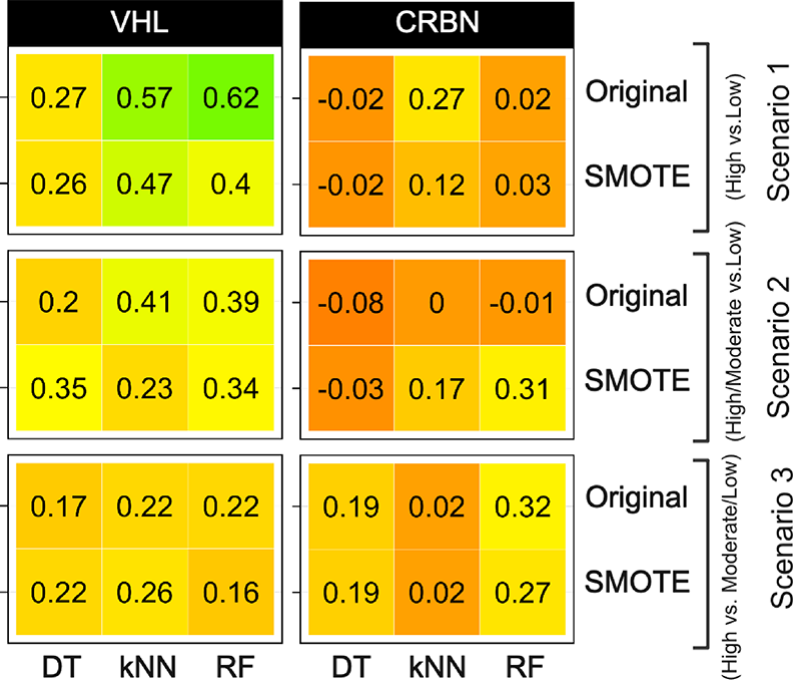
Cohen’s kappa coefficient for prediction of the permeability of the VHL and CRBN PROTACs in the blinded test set 1. The kappa coefficient is given for the three permeability scenarios for models constructed using the DT, kNN, and RF methods based on the original dataset and its SMOTE versions. Kappa coefficients have been color-coded using red-orange-yellow-green for values ranging from −0.3 to 0.7.

For the VHL PROTACs, Cohen’s kappa statistics just below and above 0.6 were obtained for prediction of high vs low permeability (scenario 1) using the kNN and RF models built using the original training set (Figure 8 and Table S6). It is interesting to note that kNN and RF classifiers performed very well in terms of predicting both the high- (sensitivity >85%) and low- (specificity >70%) permeability classes, with an impressive overall accuracy of >80% for scenario 1 (Table S6). For prediction of high/moderate vs low permeability (scenario 2), the kappa statistics were lower (approximately 0.4). Although the DT models accurately predict 74% of the lowly permeable compounds, it predicts high- and high/moderate-permeability compounds less accurately (sensitivity: 59–60%), which limits the overall accuracy to approximately 60% with a kappa value of 0.20–0.35 for scenarios 1 and 2. The overall quality of predictions for high vs moderate/low permeability (scenario 3) was rather poor (kappa: ≤0.26) owing to the high misclassification rate of the moderate/low-permeability class for all three methods (specificity: 28–74%). We note that the classification power of the RF models is higher or equal to that of the kNN classifier for all three scenarios and that RF models constructed using the SMOTE approach were worse for all three scenarios including the imbalanced scenario 2. Inferior predictions were obtained for the three permeability scenarios by the global models built using the DT, kNN, and RF classifiers after merging the VHL and CBRN datasets (Figure S8).

For CRBN, predictions of the permeabilities of the PROTACs in the blinded test set 1 were poor to fair for most models in the three scenarios, as judged by Cohen’s kappa coefficient (≤0.32, Figure 8 and Table S7). For scenarios 1 and 2, this originated from difficulties to classify the underrepresented, lowly permeable compounds correctly as all but one model had a specificity of <50% (Table S7). Thus, despite the fact that the highly permeable class is well predicted for scenarios 1 and 2 (sensitivity: ≥75% for kNN and RF), the overall quality of these models is unsatisfactory. For scenario 3, in which the high- vs moderate/low-permeability classes are better balanced, predictions using the RF model constructed using the original dataset had a sensitivity and specificity of ≥80%. In spite of the apparent high quality of the model, Cohen’s kappa coefficient (0.32) indicated it to be fair. The finding that a better model was obtained for scenario 3, which utilized a more balanced CRBN training set, provides additional support that it is the imbalanced nature of the CRBN dataset that results in the poor model performance for scenarios 1 and 2. No improvement was obtained for prediction of the CRBN PROTACs in the blinded test set 1 using the global models (Figure S8).

In summary, although models trained on the original set of PROTACs performed well on internal validation, prediction of the permeabilities varied significantly between the three scenarios and in particular between the VHL and CRBN subsets of the blinded test set 1. Excellent to satisfactory predictions were obtained in scenarios 1 and 2 for VHL using models built using the kNN and RF classifiers, while poor results were obtained for CRBN except for the RF models built for scenario 3 (Figure 8). Most likely, the main reason for the poor prediction of the blinded CRBN dataset is the uneven distribution between the permeability classes; the high-permeability class dominates the training set, while the low-permeability class is highly imbalanced. The SMOTE approach did not significantly improve predictions for the CRBN set. Presumably, SMOTE might just have amplified the noise of the minority, lowly permeable class in the training set, leading to overfitting and an inability to correctly predict the few lowly permeable PROTACs in the blinded test set 1 (Table S7). Furthermore, the SMOTE approach did not significantly improve permeability predictions for the VHL PROTACs of blinded test set 1 and was therefore not employed in the subsequent studies.

### 2.4 Retrained Classification Models

We retrained the BCMs to investigate the influence of increased size and chemical diversity of the training sets. We also hoped to minimize the impact of the unbalanced nature of the CRBN dataset and to reduce inherent bias in the dataset. To this end, the original training set and the blinded test set 1 were combined into larger retraining sets for VHL and CRBN (Figure 7), which were analyzed by PCA and compared to the corresponding original training sets (Figures S9 and S10). This comparison revealed minor shifts in the molecular property space of the three permeability classes for both types of PROTACs in addition to a more densely populated property space for the larger retraining sets. A shift in the centroid of the highly permeable class was the most pronounced difference between the original and retraining VHL sets (Figure S9). For CRBN, the orientation and shape of the ellipsoids for the low- and moderate-permeability classes indicated shifts in molecular property space (Figure S10).

New BCMs were constructed using the DT, kNN, and RF methods for the three permeability scenarios of each of the two types of PROTACs. In parallel, a second blinded test set consisting of 52 VHL and 40 CRBN PROTACs was obtained for validation of the retrained models. Just as for the first blinded test set, the permeabilities of the PROTACs in blinded test set 2 were not disclosed until after the predictions using the retrained models.

**Figure 9:**
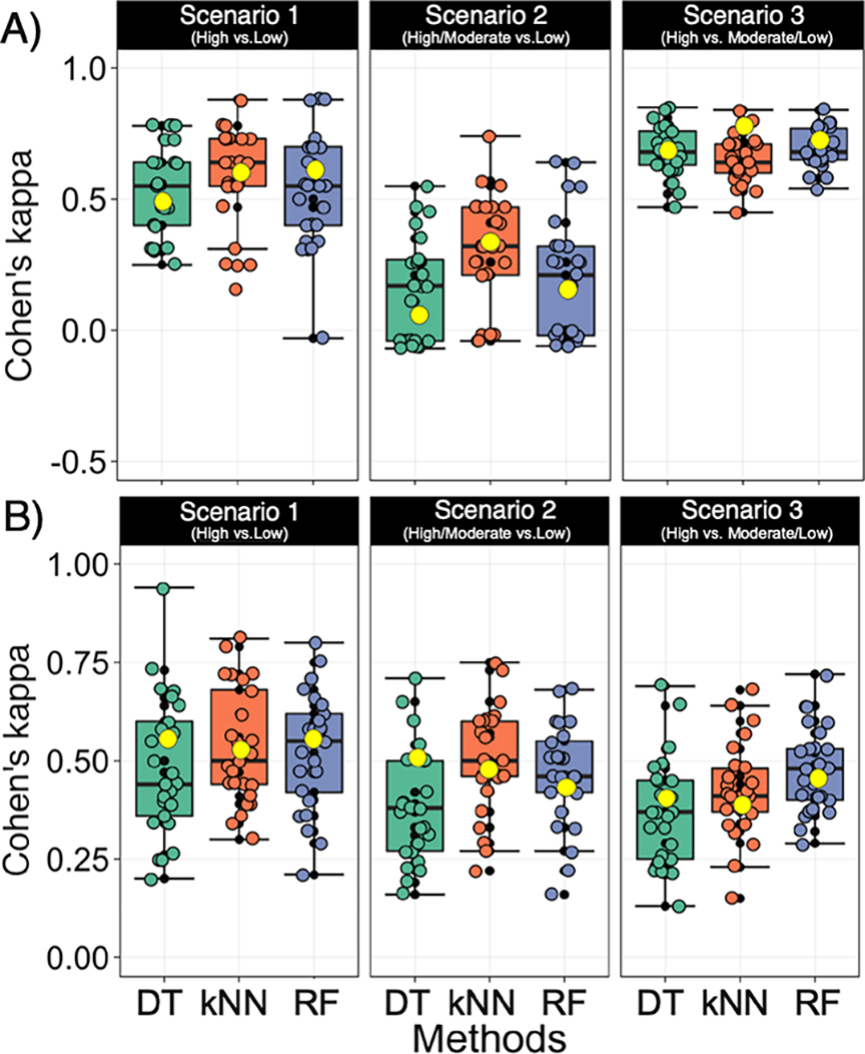
Cohen’s kappa statistics for internal validation of different retrained BCMs for three permeability scenarios of (A) CRBN and (B) VHL PROTACs in the retrained set. Box plots show the kappa values from 25 random seedlings, while the yellow circles show the kappa values from 10-fold cross validation. In the box plots, the 50^th^ percentiles are marked as horizontal bars, the 25^th^ and 75^th^ percentiles as boxes, and the 25^th^ percentile minus 1.5 × the interquartile range and the 75^th^ percentile plus 1.5 × the interquartile range as whiskers. Outliers are shown both as black dots and as circles in the color of the method used to build the model. DT: decision tree, kNN: kappa nearest neighbor, and RF: random forest. Classification models can be assessed using the following cut-offs for Cohen’s kappa: *k* < 0: no agreement, 0–0.19: poor agreement, 0.20–0.39: fair agreement, 0.40–0.59: moderate agreement, and 0.60–0.79 and 0.80–1.00: substantial to perfect agreement.^26^

Internal validation of the models obtained using the retrained sets was performed in the same way as for the models constructed using the original training set, i.e., by 25 random seedlings and 10-fold cross validation (Figure 9, Figure S11, and Table S8). For the CRBN dataset, the three machine learning methods all provided satisfactory models for the high- vs low- permeability scenario, as indicated by median kappa values of ∼0.5 (Figure 9A). With the exception of a few random splitting models, the models for the second scenario (high/moderate vs low) were much worse. Somewhat unexpectedly, the models for the third scenario yielded significantly better predictions than for scenario 2 and somewhat better than for scenario 1. Moreover, the quality of the scenario 3 models was less sensitive to the different random splits. We speculate that the improvement in the quality of the scenario 3 versus scenario 2 models originates from the fact that the moderately and lowly permeable CRBN PROTACs share similar molecular property space, which is somewhat distinct from that of the highly permeable PROTACs. The observation that the centroids of the moderately and lowly permeable CRBN PROTACs are closer to each other in the PCA of molecular property space than to the centroid of the highly permeable PROTACs lends some support to this hypothesis (Figure S10). For VHL, the three ML methods provided models of comparable quality for all three permeability scenarios (Figure 9B). However, the internal validation suggested that the models for the high vs low scenario performed slightly better than those for the two other scenarios. For the CRBN PROTACs, comparison of the models obtained using the retrained set to those of the original set showed that the retrained models were of higher quality and had lower variability in the 25 random seedling validations than the original models (cf. Figures 4 and 9). However, for VHL, the internal validation revealed that the retrained and original models were of similar quality.

**Figure 10:**
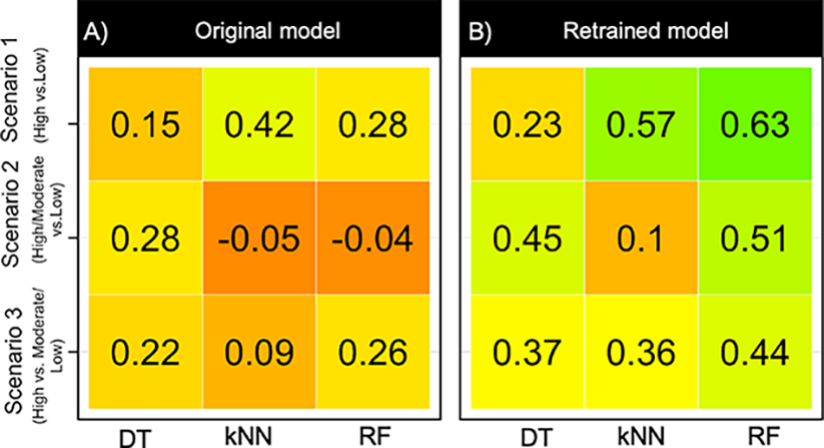
Cohen’s kappa coefficient for prediction of the permeability of the VHL PROTACs in the blinded test set 2 using models constructed with the (A) original training set and the (B) retraining set. The kappa coefficient is given for the three permeability scenarios for models constructed using the DT, kNN, and RF methods. Kappa coefficients have been color-coded using red-orange-yellow-green for values ranging from −0.30 to 0.70.

The models constructed with the DT, kNN, and RF methods using the retrained sets were employed to predict the cell permeability of the blinded test set 2 (Figure 7 and Table S9), which consisted of 52 VHL and 40 CRBN PROTACs. For comparison, the corresponding models built using the original training set were also used for prediction of the permeabilities of the blinded test set 2. Out of the nine predictions (three permeability scenarios and three methods) done for the VHL PROTACs of blinded test set 2 using the original models, only the one developed with the kNN method for the high vs low scenario performed satisfactorily (κ: 0.42, Figure 10 and Table S10). This is a significant drop in quality as compared to when the original models were used to predict scenario 1 for blinded test set 1 (κ values of 0.57 and 0.62 for kNN and RF, respectively, cf. Figure 8). For scenarios 2 and 3, the original models performed even worse in prediction of blinded test set 2 (cf. Figures 8 and 10). As will be discussed further below, these differences most likely originate in a gradual shift in the molecular property space of the three sets of VHL PROTACs (cf. comparison of datasets, below).

For VHL, the accuracy (the overall correct prediction for the two classes) of the nine predictions using the retrained models varied from 58 to 82%, with kappa statistics between 0.1 and 0.63 (Figure 10 and Table S11). It is worth to note that only the RF models performed satisfactorily (κa: >0.4) for all three permeability scenarios. For scenario 1, i.e., high vs low permeability, the RF and kNN models correctly predicted >79% of PROTACs with an impressive kappa (κ = RF: 0.63 and kNN: 0.57). Moreover, these kNN and RF models achieved a sensitivity of 75% and a specificity of 86–93%. When the moderately permeable class was combined with the highly permeable class (scenario 2), the quality of the RF model dropped somewhat (κ: 0.51) due to the fact that the specificity was reduced from 93 to 64%. For this scenario, the quality of the kNN model was poor (κ: 0.1), while DT performed well (κ: 0.45). For scenario 3 (high vs moderate/low), the DT and kNN methods performed somewhat less well (κ: < 0.4) than RF (κ: 0.44). For all three methods, this drop in quality originated from the fact that the lowly permeable class was poorly predicted, with half of the lowly permeable compounds being misclassified as highly permeable (sensitivity: 42–50%, Table S11). The likely reason for this could be that the classification models were dominated by the high/moderate-permeability compounds due to the imbalanced nature of the scenario 3 retraining set, where the low class comprised only 21% (Figure 7). Combining different classifiers to give consensus models did not improve the results (Table S12).

Although the overall accuracy of the nine predictions for the CRBN blinded test set 2 using the retrained models varied from 70 to 90%, the kappa statistics for all nine models were poor (−0.15 to 0, Table S13). The poor performance originated from the incorrect prediction of the four lowly permeable compounds in the test set by all but one model, which still only predicted one of the four lowly permeable PROTACs correctly (specificity: 0 or 25%). Thus, even though the retrained CRBN set was three times larger than the original set, its imbalance with only 6.6% lowly permeable PROTACs prevented the construction of classification models able to identify the novel low permeability in the blinded test set 2.

**Figure 11:**
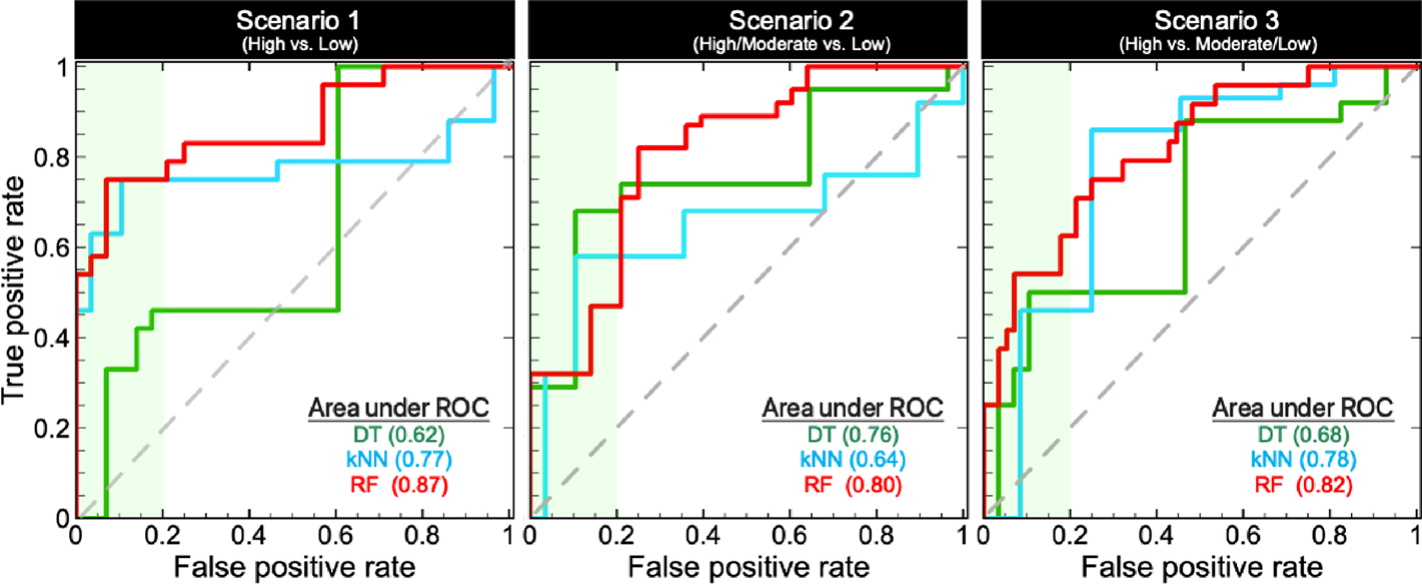
Receiver operating characteristic curve comparisons of the DT, kNN, and RF models for the three permeability scenarios of the VHL blinded test set 2. The area under the ROC curve up to a false positive rate of 0.2 (20%) is highlighted by the green region.

Receiver operating characteristic (ROC) curves are often used to evaluate the effectiveness of classification models. ROC curves provide an indication of how well a model is able to distinguish the known positives (true positive rate) from inactive compounds at various stages of database coverage.^27,28^ Consequently, ROC analyses may be used to identify early-or late-stage enrichment by models. The ROC curves for the predictions of the permeability of the VHL PROTACs in the blinded test set 2 using the retrained models demonstrate that the RF and kNN models are capable of identifying the high or high/moderate compounds with AUC values of 0.80–0.87 and 0.64–0.78, respectively, in all three scenarios (Figure 11). The DT models had somewhat lower AUC values than the kNN models. Interestingly, the RF models seem to be the most efficient, with a remarkable early enrichment in scenario 1 (80% of highly permeable compounds found after 20% of the screen).

### 2.5 Importance of Molecular Descriptors and Permeability SAR

In addition to the prediction of properties of compounds using ML algorithms, there is a growing interest in understanding the descriptors that influence the prediction, i.e., the extent to which a particular feature contributes to the prediction. Molecular descriptor contribution to a model is retrieved in the form of negative or positive coefficients, which is straightforward in many regression-like approaches.^15^ The classification approaches, on the other hand, traditionally operate as a “black box”, meaning that the features, functions, and weights provided by these models are inaccessible to interpretation. However, possibilities to extract information about the contributions of different descriptors have increasingly been incorporated in recent developments of ML algorithms, including the RF classifier.^29^

**Figure 12:**
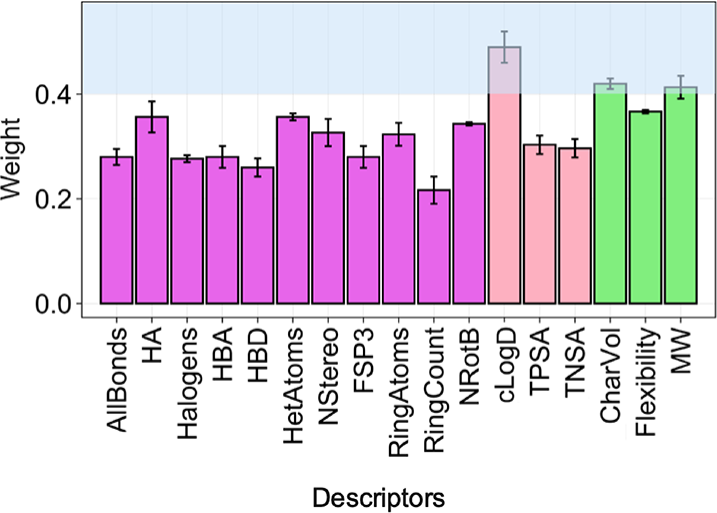
Contribution of the descriptors to the retrained RF models for prediction of the permeability of VHL PROTACs. The figure shows the mean values of the weight of each descriptor for permeability scenarios 1–3, with error bars indicating ± standard deviation. The weight of the contribution of each descriptor to the model was obtained from the 10-fold cross validation. The descriptors that contribute most to the model are indicated by the blue shading at a weight of ≥0.4. Color code: violet: countable descriptors, pink: chemical functionalities descriptors, and green: size and shape descriptors. Descriptor contributions for the individual models for scenarios 1–3 can be found in the Supporting Information, Figure S10B.

In this study, we found that the RF classifier provided the most accurate permeability models for the VHL PROTACs, whereas models for CRBN were of lower quality due to the imbalance of the datasets. Hence, descriptor contributions were retrieved from the 10-fold cross validation of the retrained RF models for VHL cell permeability (Figure S12). The most important descriptors for the three retrained models for scenarios 1–3 were lipophilicity (cLogD) and those that characterize size and shape [MW and characteristic volume (CharVol^30^), Figure 12 and Figure S12]. However, it is important to note that the contribution of the different descriptors to the model only varies by a factor of two. To some extent, this is likely a result of the fact that descriptors may be highly correlated, e.g., TPSA, HBD, and HBA, just as NRotB and flexibility. Still, the low variability in the contributions of the descriptors to the models indicates the importance of all 17 descriptors for the quality of the model and illustrates how machine learning may find patterns in complex and overlapping molecular property spaces (cf. Figure 1). It also illustrates that interpretation of ML models for design of PROTACs could remain difficult as cell permeability depends on several descriptors and not only on a few easily interpreted ones. Recent in-depth NMR and computational studies provide some support for this conclusion.^17,18^ It was found that the propensity of PROTACs to adopt folded conformations with a low solvent-accessible 3D polar surface area in an apolar environment was correlated to high cell permeability. In contrast, descriptors of lipophilicity (cLogP), size (MW), and flexibility (NRotB), which usually correlate to cell permeability, were misleading.

**Figure 13:**
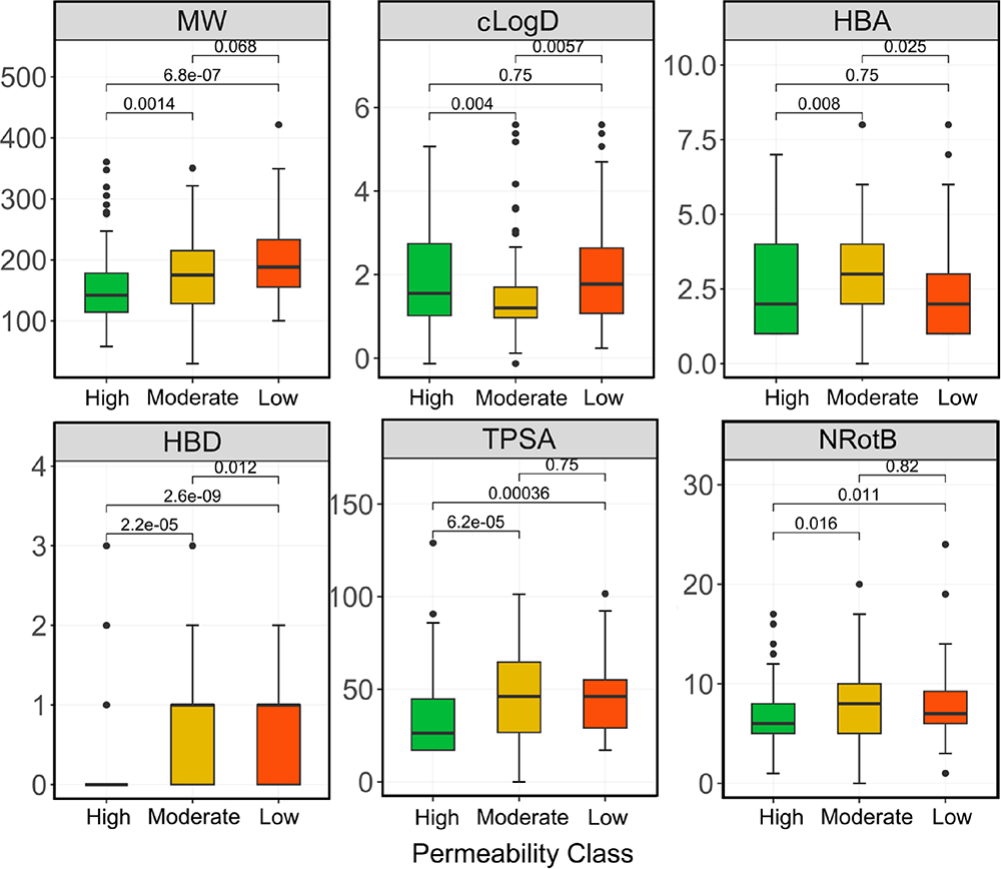
Distribution of the molecular descriptors of Lipinski’s^3^ and Veber’s^4^ guidelines for the linker part (*n* = 129) of the VHL PROTACs in the combined training set and blinded test set 1 (*n* = 253). Distributions have been calculated for the linkers of the PROTACS in each of the three permeability classes. Box plots show the 50^th^ percentiles as horizontal bars, the 25^th^ and 75^th^ percentiles as boxes, and the 25^th^ percentile minus 1.5 × the interquartile range and the 75^th^ percentile plus 1.5 × the interquartile range as whiskers. Outliers are shown both as black dots. Statistical analysis was performed using Wilcoxon’s non-parametric test.

As PROTACs consist of three domains, we investigated how the cell permeability of the VHL PROTACs was influenced by the structures of the POI ligand and the linker, both of which may allow large structural variation in the design process. Individual POI ligands and linkers were only used to assemble a few PROTACs in the training set and blinded test set 1, making it difficult to analyze the impact of a specific ligand or linker on permeability. Instead, we determined how molecular descriptors calculated for the two variable PROTAC domains correlated to cell permeability. Interestingly, the structure of the linker, the design of which is likely to allow a significant degree of variation, was found to have a somewhat larger influence on cell permeability than that of the POI ligand (Figure 13 and Figure S13). For the rather flexible linkers of the VHL PROTACs in this study (median NRotB appr. 6–8), the MW and number of HBDs had a major influence on whether or not a PROTAC displayed high or low permeability (Figure 13). TPSA, which is strongly correlated to HBDs, was also of importance. For the POI ligand, the number of HBAs and the flexibility (NRotB) were the most influential descriptors (Figure S13). We note that the descriptors of the two domains contribute differently to the PROTAC cell permeability and again conclude that it is the interplay between the properties of the overall PROTAC that determines cell permeability instead of specific properties or structural features.

### 2.6 Comparison of Datasets

**Figure 14:**
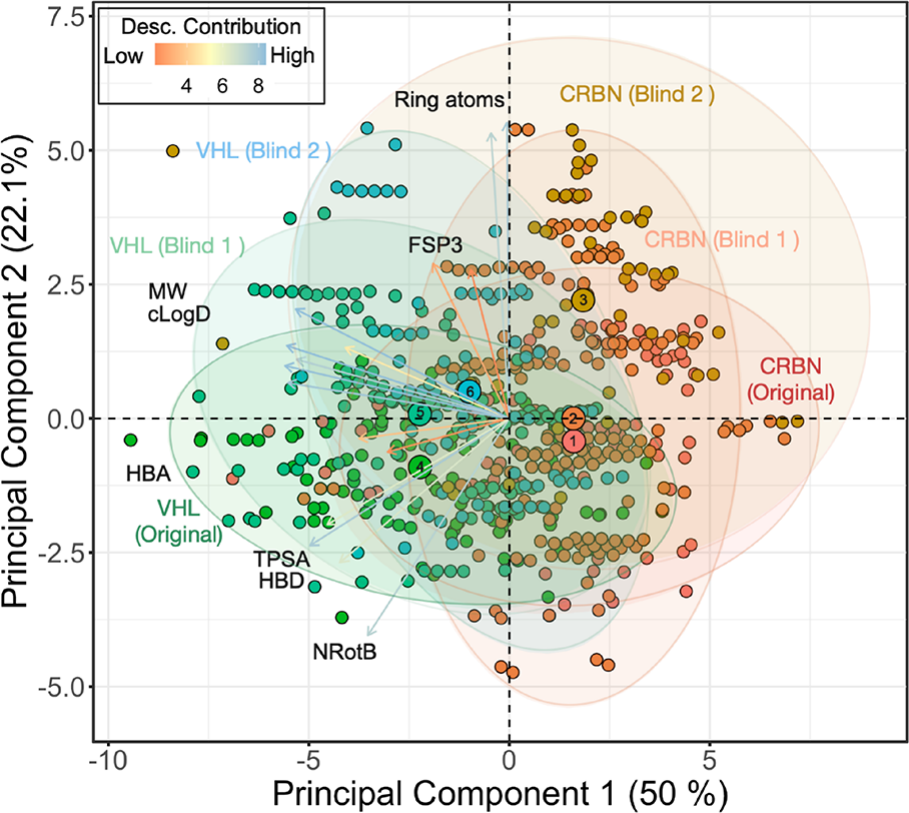
Score plot of the first two principal components from a PCA, which describes 72% of the variance for the combined original, blinded 1, and blinded 2 VHL and CRBN datasets. The PCA was based on the 17 descriptors calculated for each PROTAC, which were used for construction of the permeability models (cf. Figure 3A). Ellipses in green-blue shading show the 95% confidence intervals for the three VHL sets, while those in red-brown denote the 95% confidence intervals for the three CRBN sets. The larger, numbered circles represent the cluster centroids for each dataset. Circles numbered 1, 2, and 3 represent the original training set and blinded test sets 1 and 2, respectively, for CRBN. Circles 4–6 represent the three datasets for VHL in an identical manner. The contribution of important descriptors to the principal components is shown by an arrow, with red representing low contributions and blue representing high contributions.

The imbalanced nature of the three CRBN compound sets, which only contained a small proportion of lowly permeable PROTACs (6–10%), most likely constitutes a major reason for the poor predictability of the different CRBN models. We analyzed the three datasets, i.e., the original one and the two blinded validation sets, using PCA to understand if other reasons also contributed to the poor performance of the CRBN models (Figure 14). The three VHL datasets were analyzed in the same manner for comparison and to understand why the retrained models performed better for prediction of the blinded test set 1 than the original models. The PCA showed that the first and second principal components explained 72% of the overall variance in the datasets and that there was a significant difference within and between the VHL and CRBN datasets.

As revealed by the PCA, there is a large variation in the chemical space between the original training set and the blinded test sets 1 and 2 for the CRBN PROTACs (Figure 14). For instance, the ellipse describing the molecular property space of the compounds in the original dataset is oriented horizontally, the ellipse of blinded test set 1 is vertical, while the one of blinded test set 2 covers a spherical molecular property space. The centroids of the original dataset and blinded test set 1 are relatively close in two-dimensional property space, whereas the centroid of blinded test set 2 is located far from the other two centroids, further illustrating the differences between this set and the two other ones. Analysis of the molecular property space of the three datasets revealed a pronounced, gradual increase in cLogD, which was mirrored by a decrease in TPSA, from the original training set via the first to the second blinded test set (Figure S14). There was also a gradual increase in MW for these three sets. In addition, the blinded test set 2 was differentiated from the other two PROTAC sets by decreases in HBA and NRotB. The shift in molecular property space between the three CRBN datasets is likely to contribute to the difficulties of the BCMs to predict the permeabilities of the two blinded CRBN sets, with the second blinded test set being particularly difficult. However, as already highlighted, the highly imbalanced nature of the three CRBN sets is likely to constitute the major explanation for the poor performance of the CRBN models.

In the case of the VHL datasets, the variation in molecular property space is more subtle between the original training set and the blinded test sets 1 and 2 (Figure 14). Both the orientation and the centroids of the ellipses describing the molecular property spaces shift gradually between the three sets. Similar to the three CRBN sets, inspection of the molecular property space of the three VHL sets revealed a pronounced increase in cLogD, mirrored by a decrease in TPSA, from the original to the first and then to the second blinded test set (Figure S15 and Table S14). The reduction in TPSA originated from reductions both in the number of HBAs and HBDs. In addition, blinded test set 2 displayed minor reductions in MW and NRotB as compared to the original training set. Minor changes were also found for a few of the other descriptors, i.e., CharVol, RingAtoms, halogens, and ring count (Table S14). Overall, the more gradual shifts in molecular property space between the three datasets, in combination with their more balanced nature, constitute the basis for the success of the BCMs in predicting the cell permeability of the VHL PROTACs in blinded test sets 1 and 2.

For the three VHL sets, the gradual shift in molecular property space, illustrated by the increase in cLogD and the decrease in TPSA, was accompanied by an increase in the proportion of highly permeable PROTACs from 40% for the original training set to 53 and 46% for blinded test sets 1 and 2, respectively (Figure S16). Further investigation of the properties of the PROTACs in the three sets revealed that the gradual increase in cLogD originated both from projects contributing to more than one of the three sets and from initiation of projects involving more lipophilic PROTACs. The decrease in TPSA between the original and blinded test set 1 originated from projects present in both sets, while the decrease from the first to the second blinded test set mainly resulted from addition of new projects into the portfolio. It thus appears that the increased proportion of highly permeable VHL PROTACs originates both from improved design strategies within projects and from an evolution of the project portfolio.

## 3 Summary and Conclusions

We have used four state-of-the-art machine learning methods, i.e., random forest, decision tree, support vector machine, and kappa nearest neighbor to construct binary classification models for prediction of the cell permeability of CRBN and VHL PROTACs. The models were constructed using training sets of 113 CRBN and 115 VHL PROTACs, for which the cell permeability had been determined and classified as high, moderate, or low. We emphasize that there was no clear separation of chemical space between the three permeability classes within the two sets of PROTACs; instead, they largely occupied the same chemical space. Therefore, BCMs were constructed for three permeability scenarios: the first used only the highly and lowly permeable compounds, while the moderate class was merged with either the high or low class in scenarios 2 and 3, respectively. As the CRBN dataset was highly imbalanced with very few lowly permeable PROTACs, the SMOTE technique, which duplicates the minority class, was used in attempts to improve the permeability models. Each model was constructed using a set of 17 molecular descriptors, which are used to assess “drug-likeness” in drug discovery projects, and that were calculated from the SMILES structures of the PROTACs. After internal validation, the models were employed to predict the permeabilities of test sets of CRBN (*n* = 236) and VHL (*n* = 138) PROTACs, the cell permeabilities of which were kept blinded until after the predictions. Retrained models were built after combination of the original and the first blinded test set. Then, the ability of the retrained models to predict the permeabilities of a second blinded test set of PROTACs (40 CRBN and 52 VHL) was determined and compared to the predictivity of the models constructed using the original training set.

For the VHL PROTACs, we found that high-quality BCMs could be constructed with the RF and kNN classifiers for the prediction of high versus low cell permeability (scenario 1). These models predicted the permeabilities for the first blinded test set with high accuracies (80%) and excellent kappa statistics (κ appr. 0.6). RF and kNN models for scenario 2 were of moderate quality (appr. 75% accuracy, κ appr. 0.4) when applied to the blinded test set 1, while those for scenario 3 were much inferior (appr. 50% accuracy, κ appr. 0.2). For the second blinded test set of VHL PROTACs, permeability predictions improved when the models had been retrained by combining the original training set with the first blinded test set as compared to when the original models were used. For instance, the retrained RF and kNN models had accuracies of ≥79% and high kappa statistics (κ appr. 0.6) for scenario 1, i.e., prediction of high vs low permeability, whereas the original RF and kNN models performed less well for blinded test set 2 (66 and 74% accuracies, κ = 0.28 and 0.42, respectively). The reason for the improvement was traced to a gradual shift in molecular property space that provided a larger and more diverse training set, which increased the applicability domain of the retrained models as compared to the original ones.

High-quality, predictive BCMs could not be generated for the CRBN PROTACs in spite of significant effort. This difficulty originated mainly from the highly imbalanced nature of the three CRBN datasets, which contained only 7–8% lowly permeable PROTACs as compared to 17–40 and 52–76% having moderate and high permeability, respectively. In addition, the shift in molecular property space between the three CRBN datasets is likely to contribute to the difficulties of the BCMs to predict the permeabilities of the two blinded CRBN sets. Use of the SMOTE technique, which duplicates the minority class, in attempts to reduce the misclassification rate in imbalanced classification models was not successful.

There is a growing interest in understanding the descriptors that influence the prediction of machine learning models, i.e., the extent to which particular structural features and properties contribute to the prediction. However, all 17 descriptors that were used to build the BCMs were found to be important for the VHL models for the three permeability scenarios, even though a few (cLogD, MW, and CharVol) had somewhat higher weight. This also agrees with the fact that we observed a correlation between the proportion of highly permeable VHL PROTACs and an increase in cLogD, paralleled by a decrease in TPSA, between the three sets of VHL PROTACs. Our results illustrate that machine learning can find patterns in complex molecular property space in situations when multiple descriptors contribute to the investigated property, i.e., cell permeability in this case. The results also indicate that it may not always be possible to construct models that are easy to interpret in terms of which particular structural features should be adjusted in the design of the next set of compounds. One reason for this complex behavior may be the 3D folding of PROTACs. Recent studies have revealed that the propensity of PROTACs to adopt folded, non-polar conformations in an apolar environment correlates to high cell permeability.^17,18^

We conclude that BCMs can be implemented to support decision-making in drug discovery projects so that PROTACs predicted to have high permeability are prioritized for synthesis and subsequent testing over those predicted to have low permeability. Such classification models could be of particular value for prioritization of compounds early in lead optimization before large numbers of PROTACs have been synthesized in the project. As the PROTACs we used to construct the models overlap with the chemical space of >1800 publicly available PROTACs, models constructed as described in this manuscript are likely to have wide applicability. However, it is important that balanced training sets are used and that models are not employed outside their applicability domain. It should be noted that relatively few descriptors (17) that were rapidly calculated from the SMILES structures of the PROTACs were used to describe the compounds in the training sets used herein. This raises the possibility that even more accurate models, and perhaps models that allow multiclass classification and that provide facile interpretation for the next rounds of design, can be built if larger sets of descriptors and/or more informative descriptors are used. The modular construction of PROTACs facilitates the synthesis of large numbers of compounds, which can be profiled rapidly using in vitro assay. Access to larger training sets ADMET data than those available to us is also likely to improve the quality of models built by machine learning. We are therefore hopeful that machine learning will be of major importance in capitalizing on this data so that orally available PROTACs can be discovered and brought into clinical studies.

## 4 Methods

### 4.1 Compounds

All PROTACs were prepared at Bayer AG, and their structures were confirmed by high-resolution mass spectrometry and ^1^H NMR spectroscopy. Purities were determined by LCMS (Table 2).

**Table 2:** Overview of Purities of the PROTACs Included in the Training and Tests Sets

	CRBN, *n* = 389 (%)	VHL, *n* = 305 (%)
median	95	94.5
lower quartile	93	92
upper quartile	97	99
minimum	75	76
maximum	100	100
standard deviation	4.72	4.71

### 4.2 Molecular Descriptors

A set of 17 molecular descriptors that represent molecular size, shape, countables, and chemical functionalities were calculated from the SMILES structure of each PROTAC using Pipeline Pilot (version 16.5.0.143)^31^ with built-in property calculation functions (Supporting Information Table S1). The lipophilicity descriptor (clogD) was predicted using a multitask model trained with Bayer data on multiple physicochemical endpoints.^32^ Characteristic volume (CharVol) was calculated in Pipeline Pilot following the formula from Abraham and McGowan.^30^ Subsequently, descriptors were normalized with decimal scaling as implemented in KNIME (4.2.3).^33^

### 4.3 Determination of Cell Permeability

The ratio between the potencies for binding of a PROTAC to its E3 ligase in a cell-based and in a biochemical assay is the measure of cell permeability used in this investigation.^19^ The potencies were determined as reported previously for binding to VHL^17^ and CRBN,^18^ respectively.

Permeability class boundaries for the VHL and CRBN PROTACs were derived from the observation that the majority of compounds that display a strong drop-off, i.e., a high numerical value for the permeability ratio, show cellular IC_50_ values where their biological usefulness is questionable. For instance, for VHL-based PROTACs, ≥90% of the compounds with a permeability ratio of >40 (the cut-off for low permeability) show a cellular IC_50_ value of >10 μM. Conversely, the observation was made that the majority of compounds that display a weak drop-off, i.e., a low numerical value for the permeability ratio, show cellular IC_50_ values where biological usefulness comes within reach. Thus, for VHL-based PROTACs, ≥90% of the compounds with a permeability ratio of <16 (the cut-off for high permeability) show a cellular IC_50_ of <10 μM.

The differences in permeability class boundaries between VHL and CRBN go back to the observation that CRBN-based PROTACs less often show low permeabilities (high numerical values for the permeability ratio) and less often display cellular IC_50_ values >10 μM compared to VHL-based PROTACs. To reflect these trends, permeability class boundaries for CRBN-based PROTACs were adjusted. Thus, the cut-off for “low” permeability was reduced to >25, while the cut-off for “high” permeability was reduced to <6. Use of the boundary of >25 for low permeability resulted in the fact that ≥90% of the CRBN-based PROTACs displayed a cellular IC_50_ value of >5 μM.

### 4.4 Imbalanced Datasets

One of the most common problems with high-throughput screening and most industrial drug discovery projects (in terms of data modeling) is imbalanced datasets, i.e., that the proportion of data points for each class varies significantly,^34^ which may result in inaccurate or biased predictions when using conventional classification algorithms. Several resampling techniques have been used to reduce the misclassification rate in imbalanced classification models, including the SMOTE technique (synthesis of minority oversampling technique), which duplicates the minority class using the kappa nearest neighbor methodology.^24^ Unlike under-sampling, which results in information loss by discarding a substantial proportion of the data from the majority class, oversampling procedures increase the number of data points in the minority class, while preserving the majority class observations that are unchanged. Oversampling, on the other hand, often requires more training time and may result in overfitting.^35^ In the current datasets, the “low”-permeability class is under-sampled (7–25%) as compared to the “high”- (46–59%) and “moderate”- (44–45%) permeability classes.

### 4.5 Selection of Training and Internal Test Sets

The data set was divided into training and test sets using the stratified sampling approach with an 80:20 ratio. Stratified sampling distributes each permeability class evenly across the training and test sets and is especially beneficial for imbalanced datasets. To avoid chance correlations and improve reproducibility, 25 random stratified sampling seedlings were used.

In addition to training and test set validation, all models were cross-validated with 10-fold cross validation. All cross validations were carried out with the “X-partitioner” procedure as implemented in KNIME (4.2.3).

### 4.6 Construction of Machine Learning Models

The machine learning methods used in this study, i.e., random forest, decision tree, support vector machine, and kappa nearest neighbor, are based on different concepts and are considered as state-of-art for classification modeling.^23^ All the BCMs and data analyses reported in this study were constructed using KNIME (4.2.3) and Weka data mining software^36^ (cf. Supporting Information Figure S4 and Table S2). Plots were made using RStudio and Origin. A Microsoft Windows 10, 64-bit based desktop computer, 8-core i7-6700K CPU@ 4.00GHz with NVIDIA GeForce GTX980 Ti GPU was used.

### 4.7 Model Assessment

All classification models were assessed using Cohen’s kappa (eq 1) based on the test set (stratified sampling of 80:20 ratio of training and test set with 25 times random splitting) and 10-fold cross validation sets. In addition, the quality of each model was evaluated based on its sensitivity (eq 2), specificity (eq 3), balanced prediction (eq 4), and overall accuracy (eq 5).

Cohen’s kappa is a measure of the agreement between two classes that takes both correct and misclassification rates into consideration.
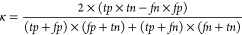
1where *tp* are the true positives, *fp* are the false positives, *tn* are the true negatives, and *fn* are the false negatives.

Sensitivity is the correct prediction of positive class.
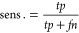
2

Specificity is the correct prediction of negative class.
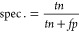
3

Balanced prediction is the balanced prediction between the negative and positive class.
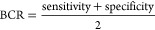
4

Accuracy is the overall correct prediction of both positive and negative classes.
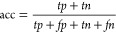
5

ROC-AUC is defined as ROC curves that plot sensitivity versus 1 – specificity (also known as false positive rate or ) at different probability thresholds to separate positive from negative class, e.g., the probability of prediction **≥**0.5 and <0.5 on a 0–1 probability scale is considered as active and inactive, respectively. The AUC measures a classifier’s ability to distinguish between positive and negative classes and summarizes the ROC curve (AUC).^28^

Limits for model assessment using Cohen’s kappa include the following: κ < 0: no agreement, 0–0.19: poor agreement, 0.20–0.3: fair agreement, 0.40–0.59 moderate agreement, 0.60–0.79: substantial agreement, and 0.80–1.00: perfect agreement.^26^ In general, models having a Cohen’s kappa of >0.4 are considered to provide satisfactory predictions in machine learning binary classifications.^23,26,37^

### 4.8 Applicability Domain

An AD experiment was performed to assess the reliability of the models for prediction of the permeability of new compounds. AD generates the first structural warning for the data set and is primarily used to determine if a new molecular entity is present in the training set’s chemical domain.^38−40^ Several AD methods have been introduced, each with its own set of benefits and drawbacks. Descriptor “ranges”, “Euclidean distances (ED)”, and “probability density” are three well-known AD approaches. In the present study, the ED method was used as implemented in the Ambit discovery software.^41^ To remove collinearities among the descriptors, Ambit discovery uses PCA to preprocess the given dataset. The AD is then calculated for both the internal and external test sets using the ED approach.

## Supporting Information Available

Molecular descriptors, workflows and settings for construction of ML methods, statistics for ML models, and lists of compounds in training and test sets (PDF)

## Abbreviations

AD: applicability domain
BCM: binary classification model
bRo5: beyond rule of 5
BTK: Bruton’s tyrosine kinase
CRBN: cereblon
DT: decision tree
kNN: kappa nearest neighbor
LPE: lipophilic permeability efficiency
ML: machine learning
NRotB: number of rotatable bonds
RF: random forest
SMOTE: synthesis of minority oversampling technique
PCA: principal component analysis
POI: protein of interest
PROTAC: proteolysis targeting chimera
ROC: receiver operating characteristic
SVM: support vector machine
TPSA: topological polar surface area
VHL: von Hippel–Lindau

## References

[ref1] Békés M.; Langley D. R.; Crews C. M. PROTAC targeted protein degraders: the past is prologue. Nat. Rev. Drug Disc. 2022, 21, 181–200. 10.1038/s41573-021-00371-6. PMC8765495 35042991

[ref2] Weng G.; Shen C.; Cao D.; Gao J.; Dong X.; He Q.; Yang B.; Li D.; Wu J.; Hou T. PROTAC-DB: an online database of PROTACs. Nucleic Acids Res. 2021, 49, D1381–D1387. 10.1093/nar/gkaa807. 33010159 PMC7778940

[ref3] Lipinski C. A.; Lombardo F.; Dominy B. W.; Feeney P. J. Experimental and computational approaches to estimate solubility and permeability in drug discovery and development settings. Adv. Drug Delivery Rev. 2001, 46, 3–26. 10.1016/S0169-409X(00)00129-0. 11259830

[ref4] Veber D. F.; Johnson S. R.; Cheng H.-Y.; Smith B. R.; Ward K. W.; Kopple K. D. Molecular properties that influence the oral bioavailability of drug candidates. J. Med. Chem. 2002, 45, 2615–2623. 10.1021/jm020017n. 12036371

[ref5] Edmondson S. D.; Yang B.; Fallan C. Proteolysis targeting chimeras (PROTACs) in ‘beyond rule-of-five’ chemical space: Recent progress and future challenges. Bioorg. Med. Chem. Lett. 2019, 29, 1555–1564. 10.1016/j.bmcl.2019.04.030. 31047748

[ref6] Maple H. J.; Clayden N.; Baron A.; Stacey C.; Felix R. Developing degraders: principles and perspectives on design and chemical space. MedChemCommun. 2019, 10, 1755–1764. 10.1039/C9MD00272C. PMC6894040 31867093

[ref7] Poongavanam V.; Kihlberg J. PROTAC cell permeability and oral bioavailability: a journey into uncharted territory. Future Med. Chem. 2022, 14, 123–126. 10.4155/fmc-2021-0208. 34583518

[ref8] Doak B. C.; Over B.; Giordanetto F.; Kihlberg J. Oral druggable space beyond the rule of 5: Insights from drugs and clinical candidates. Chem. Biol. 2014, 21, 1115–1142. 10.1016/j.chembiol.2014.08.013. 25237858

[ref9] DeGoey D. A.; Chen H.-J.; Cox P. B.; Wendt M. D. Beyond the rule of 5: lessons learned from AbbVie’s drugs and compound collection. J. Med. Chem. 2018, 61, 2636–2651. 10.1021/acs.jmedchem.7b00717. 28926247

[ref10] García Jiménez D.; Rossi Sebastiano M.; Vallaro M.; Mileo V.; Pizzirani D.; Moretti E.; Ermondi G.; Caron G. Designing soluble PROTACs: Strategies and preliminary guidelines. J. Med. Chem. 2022, 65, 12639–12649. 10.1021/acs.jmedchem.2c00201. 35469399 PMC9574862

[ref11] Klein V. G.; Townsend C. E.; Testa A.; Zengerle M.; Maniaci C.; Hughes S. J.; Chan K.-H.; Ciulli A.; Lokey R. S. Understanding and improving the membrane permeability of VH032-based PROTACs. ACS Med. Chem. Lett. 2020, 11, 1732–1738. 10.1021/acsmedchemlett.0c00265. 32939229 PMC7488288

[ref12] Klein V. G.; Bond A. G.; Craigon C.; Lokey R. S.; Ciulli A. Amide-to-ester substitution as a strategy for optimizing PROTAC permeability and cellular activity. J. Med. Chem. 2021, 64, 18082–18101. 10.1021/acs.jmedchem.1c01496. 34881891 PMC8713283

[ref13] Hendrick C. E.; Jorgensen J. R.; Chaudhry C.; Strambeanu I. I.; Brazeau J.-F.; Schiffer J.; Shi Z.; Venable J. D.; Wolkenberg S. E. Direct-to-biology accelerates PROTAC synthesis and the evaluation of linker effects on permeability and degradation. ACS Med. Chem. Lett. 2022, 12, 1182–1190. 10.1021/acsmedchemlett.2c00124. PMC9290060 35859867

[ref14] Zhang J.; Che J.; Luo X.; Wu M.; Kan W.; Jin Y.; Wang H.; Pang A.; Li C.; Huang W.; Zeng S.; Zhuang W.; Wu Y.; Xu Y.; Zhou Y.; Li J.; Dong X. Structural feature analyzation strategies toward discovery of orally bioavailable PROTACs of Bruton’s tyrosine kinase for the treatment of lymphoma. J. Med. Chem. 2022, 65, 9096–9125. 10.1021/acs.jmedchem.2c00324. 35671249

[ref15] Over B.; Matsson P.; Tyrchan C.; Artursson P.; Doak B. C.; Foley M. A.; Hilgendorf C.; Johnston S.; Lee I.; Lewis R.; McCarren P.; Muncipinto G.; Norinder U.; Perry M.; Duvall J. R.; Kihlberg J. Structural and conformational determinants of macrocycle cell permeability. Nat. Chem. Biol. 2016, 12, 1065–1074. 10.1038/nchembio.2203. 27748751

[ref16] Poongavanam V.; Atilaw Y.; Ye S.; Wieske L. H. E.; Erdelyi M.; Ermondi G.; Caron G.; Kihlberg J. Predicting the permeability of macrocycles from conformational sampling – limitations of molecular flexibility. J. Pharm. Sci. 2021, 110, 301–313. 10.1016/j.xphs.2020.10.052. 33129836

[ref17] Atilaw Y.; Poongavanam V.; Svensson Nilsson C.; Nguyen D.; Giese A.; Meibom D.; Erdelyi M.; Kihlberg J. Solution conformations shed light on PROTAC cell permeability. ACS Med. Chem. Lett. 2021, 12, 107–114. 10.1021/acsmedchemlett.0c00556. 33488971 PMC7812666

[ref18] Poongavanam V.; Atilaw Y.; Siegel S.; Giese A.; Lehmann L.; Meibom D.; Erdelyi M.; Kihlberg J. Linker-Dependent Folding Rationalizes PROTAC Cell Permeability. J. Med. Chem. 2022, 65, 13029–13040. 10.1021/acs.jmedchem.2c00877. 36170570 PMC9574858

[ref19] Shah R. R.; Redmond J. M.; Mihut A.; Menon M.; Evans J. P.; Murphy J. A.; Bartholomew M. A.; Coe D. M. Hi-JAK-ing the ubiquitin system: The design and physicochemical optimisation of JAK PROTACs. Bioorg. Med. Chem. 2020, 28, 115326 10.1016/j.bmc.2020.115326. 32001089

[ref20] Schneider P.; Walters W. P.; Plowright A. T.; Sieroka N.; Listgarten J.; Goodnow R. A. Jr.; Fisher J.; Jansen J. M.; Duca J. S.; Rush T. S.; Zentgraf M.; Hill J. E.; Krutoholow E.; Kohler M.; Blaney J.; Funatsu K.; Luebkemann C.; Schneider G. Rethinking drug design in the artificial intelligence era. Nat. Rev. Drug Discovery 2020, 19, 353–364. 10.1038/s41573-019-0050-3. 31801986

[ref21] Aleksić S.; Seeliger D.; Brown J. B. ADMET predictability at Boehringer Ingelheim: state-of-the-art, and do bigger datasets or algorithms make a aifference?. Mol. Inf. 2022, 41, 2100113 10.1002/minf.202100113. 34473408

[ref22] Cáceres E. L.; Tudor M.; Cheng A. C. Deep learning approaches in predicting ADMET properties. Future Med. Chem. 2020, 12, 1995–1999. 10.4155/fmc-2020-0259. 33124448

[ref23] Vasanthanathan P.; Taboureau O.; Oostenbrink C.; Vermeulen N. P.; Olsen L.; Jorgensen F. S. Classification of cytochrome P450 1A2 inhibitors and noninhibitors by machine learning techniques. Drug Metab. Dispos. 2009, 37, 658–664. 10.1124/dmd.108.023507. 19056915

[ref24] Chawla N. V.; Bowyer K. W.; Hall L. O.; Kegelmeyer W. P. SMOTE: Synthetic minority over-sampling technique. J. Artif. Intell. Res. 2002, 16, 321–357. 10.1613/jair.953.

[ref25] Batista G. E. A. P. A.; Prati R. C.; Monard M. C. A study of the behavior of several methods for balancing machine learning training data. SIGKDD Explor. Newsl. 2004, 6, 20–29. 10.1145/1007730.1007735.

[ref26] Chohan K. K.; Paine S. W.; Mistry J.; Barton P.; Davis A. M. A rapid computational filter for cytochrome P450 1A2 inhibition potential of compound libraries. J. Med. Chem. 2005, 48, 5154–5161. 10.1021/jm048959a. 16078835

[ref27] Triballeau N.; Acher F.; Brabet I.; Pin J. P.; Bertrand H. O. Virtual screening workflow development guided by the ″receiver operating characteristic″ curve approach. Application to high-throughput docking on metabotropic glutamate receptor subtype 4. J. Med. Chem. 2005, 48, 2534–2547. 10.1021/jm049092j. 15801843

[ref28] Bradley A. P. The use of the area under the roc curve in the evaluation of machine learning algorithms. Pattern Recognit. 1997, 30, 1145–1159. 10.1016/S0031-3203(96)00142-2.

[ref29] Jiménez-Luna J.; Grisoni F.; Schneider G. Drug discovery with explainable artificial intelligence. Nat. Mach. Intell. 2020, 2, 573–584. 10.1038/s42256-020-00236-4.

[ref30] Abraham M. H.; McGowan J. C. The use of characteristic volumes to measure cavity terms in reversed phase liquid chromatography. Chromatographia 1987, 23, 243–246. 10.1007/BF02311772.

[ref31] Pipeline Pilot version 16.5.0.143; Dassault Systemes BIOVIA Corp.: San Diego, CA: 2016.

[ref32] Montanari F.; Kuhnke L.; Ter Laak A.; Clevert D. A. Modeling physico-chemical ADMET endpoints with multitask graph convolutional networks. Molecules 2020, 25, 44 10.3390/molecules25010044. PMC6982787 31877719

[ref33] Berthold M. R.; Cebron N.; Dill F.; Gabriel T. R.; Kötter T.; Meinl T.; Ohl P.; Thiel K.; Wiswedel B. KNIME - the Konstanz information miner: version 2.0 and beyond. SIGKDD Explor. Newsl. 2009, 11, 26–31. 10.1145/1656274.1656280.

[ref34] Zakharov A. V.; Peach M. L.; Sitzmann M.; Nicklaus M. C. QSAR modeling of imbalanced high-throughput screening data in PubChem. J. Chem. Inf. Model. 2014, 54, 705–712. 10.1021/ci400737s. 24524735 PMC3985743

[ref35] Branco P.; Torgo L.; Ribeiro R. P. A Survey of predictive modeling on imbalanced domains. ACM Comput. Surv. 2016, 49, 31 10.1145/2907070.

[ref36] Hall M.; Frank E.; Holmes G.; Pfahringer B.; Reutemann P.; Witten I. H. The WEKA data mining software: an update. SIGKDD Explor. Newsl. 2009, 11, 10–18. 10.1145/1656274.1656278.

[ref37] Margherita G. E., Bagli; Giorgio V. Metrics for multi-class classification: an overview. arXiv preprint 2020, arXiv:2008.05756.

[ref38] Netzeva T. I.; Worth A. P.; Aldenberg T.; Benigni R.; Cronin M. T. D.; Gramatica P.; Jaworska J. S.; Kahn S.; Klopman G.; Marchant C. A.; Myatt G.; Nikolova-Jeliazkova N.; Patlewicz G. Y.; Perkins R.; Roberts D. W.; Schultz T. W.; Stanton D. T.; van de Sandt J. J. M.; Tong W. D.; Veith G.; Yang C. H. Current status of methods for defining the applicability domain of (quantitative) structure-activity relationships - The report and recommendations of ECVAM Workshop 52. Atla-Altern Lab. Anim. 2005, 33, 155–173. 10.1177/026119290503300209. 16180989

[ref39] Weaver S.; Gleeson N. P. The importance of the domain of applicability in QSAR modeling. J. Mol. Graphics Modell. 2008, 26, 1315–1326. 10.1016/j.jmgm.2008.01.002. 18328754

[ref40] Mathea M.; Klingspohn W.; Baumann K. Chemoinformatic classification methods and their applicability domain. Mol. Inf. 2016, 35, 160–180. 10.1002/minf.201501019. 27492083

[ref41] Jaworska J.; Nikolova-Jeliazkova N. How can structural similarity analysis help in category formation?. SAR QSAR Environ. Res. 2007, 18, 195–207. 10.1080/10629360701306050. 17514565

