# Supplementary material for: Predictive Modeling of PROTAC Cell Permeability with Machine Learning
Source: ACS Omega. 2023 Feb 1;8(6):5901–16. doi: 10.1021/acsomega.2c07717 (PMC9933238; doi:10.1021/acsomega.2c07717)
Supplement: Supplementary file 1 — ao2c07717_si_001.pdf [file ao2c07717_si_001.pdf]

SUPPORTING INFORMATION

for

Predictive Modeling of PROTAC Cell Permeability with Machine Learning

Vasanthanathan Poongavanam, [a,]* Florian Kölling,[b] Anja Giese,[c] Andreas H. Göller,[b] Lutz Lehmann,[d] Daniel Meibom[d] and Jan Kihlberg[a,]*

[a] Department of Chemistry-BMC, Box 576, Uppsala University, SE-75123 Uppsala, Sweden

[b] Computational Molecular Design, Bayer AG, 42096, Wuppertal, Germany

[c] Drug Discovery Sciences, Bayer AG, 13342 Berlin, Germany

[d] Drug Discovery Sciences, Bayer AG, 42113, Wuppertal, Germany

Table of Contents

Figure S1. Calculated molecular descriptors by permeability class (VHL original training set)....3

Figure S2. Calculated molecular descriptors by permeability class (CRBN original training set)....4

Table S1. List of the molecular descriptors used to build models....5

Figure S3. Comparison of molecular descriptors for PROTAC sets....6

Figure S4. Overall KNIME workflow....7

Table S2. Settings used for machine learning methods....8

Table S3. Summary of "CfsSubsetEval-BestFirst"-based models (VHL dataset/10-fold CV)[a]....9

Table S4. Compounds used for model training and cross-validation by permeability class scenario....9

Figure S5. Summary of Cohan's Kappa statistics for internal validation of the global classification models constructed by combination of the VHL and CRBN training sets....10

Figure S6. Accuracies for CRBN (A) and VHL (B) models constructed using the original training sets....11

Figure S7. Applicability domain assessment....12

Table S5. Number of compounds by permeability class scenario for blinded test set 1[a]....12

Table S6. Summary of blinded predictions of VHL PROTACs in blinded test set 1..........13
Figure S8. Cohen's kappa coefficient for prediction of the permeability of the VHL and CRBN PROTACs in the blinded test set 1 using the global classification models 14
Table S7. Summary of blinded predictions of CRBN PROTACs in blinded test set 1.......15
Figure S9. Score plots of the first two principal components from PCAs of the original and retrained VHL datasets 16
Figure S10. Score plots of the first two principal components from PCAs of the original and retrained CRBN datasets 16
Figure S11. Accuracies from internal test set validations of retrained CRBN (A) and VHL (B) models...17
Table S8. Summary of 10-fold cross validation of retrained BCMs.[a] 18
Table S9. Number of compounds by permeability class scenario for blinded test set 2[a] 18
Table S10. Performance of original models on permeability predictions of VHL blinded test set 2.[a] 19
Table S11. Performance of retrained models on permeability predictions of VHL blinded test set 2.[a] 19
Table S12. Consensus predictions for permeability scenarios of the VHL blinded test set 2 using the retrained models 20
Table S13. Performance of retrained models on permeability predictions of CRBN blinded test set 2.[a] 21
Figure S12. Descriptor importance for retrained VHL and CRBN models........................22
Figure S13. Distribution of the molecular descriptors of Lipinski's and Veber's guidelines for the POI ligand part (n=46) of the VHL PROTACs in the training set and blinded test set 1 (n=253) 24
Figure S14. Molecular descriptor distribution for the three CRBN datasets.......................25
Figure S15. Molecular descriptor distribution for the three VHL datasets.........................26
Table S14. Distribution of all 17 molecular descriptors for the three VHL datasets...........27
Figure S16. Distribution of the PROTACs by permeability class for the three VHL datasets 27
References *28*

Figure S1. Calculated molecular descriptors by permeability class (VHL original training set)

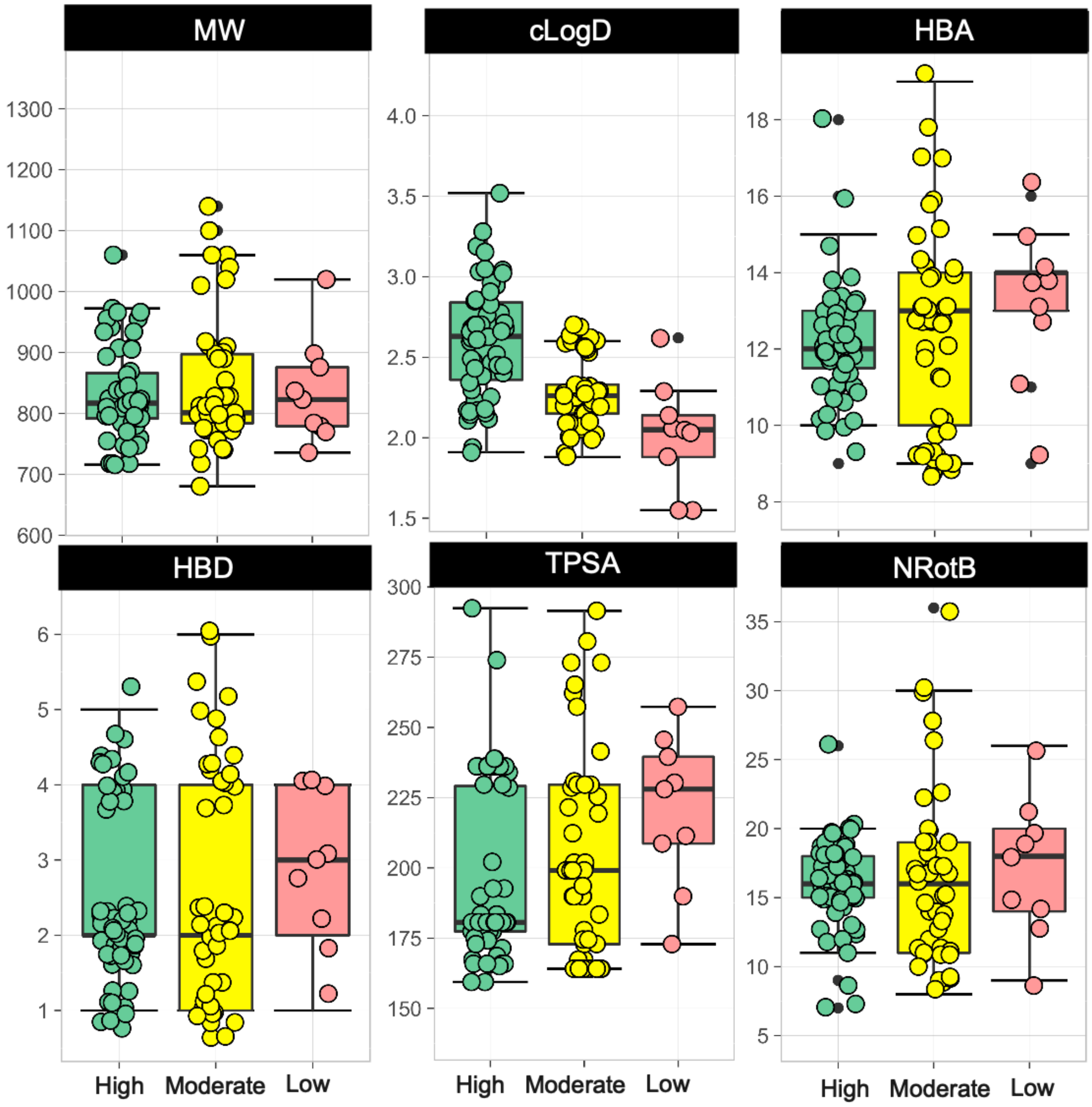

Abbreviations: Molecular weight (MW, Da), lipophilicity (cLogD), hydrogen bond acceptors and donors (HBA and HBD), topological polar surface area (TPSA, Å^2), and number of rotatable bonds (NRotB).

Box plots show the 50th percentiles as horizontal bars, the 25th and 75th percentiles as boxes, the 25th percentile minus 1.5 x the interquartile range and the 75th percentile plus 1.5 x the interquartile range as whiskers for the PROTACs. Outliers are shown both as black dots and as circles in the color of the appropriate descriptor.

Figure S2. Calculated molecular descriptors by permeability class (CRBN original training set).

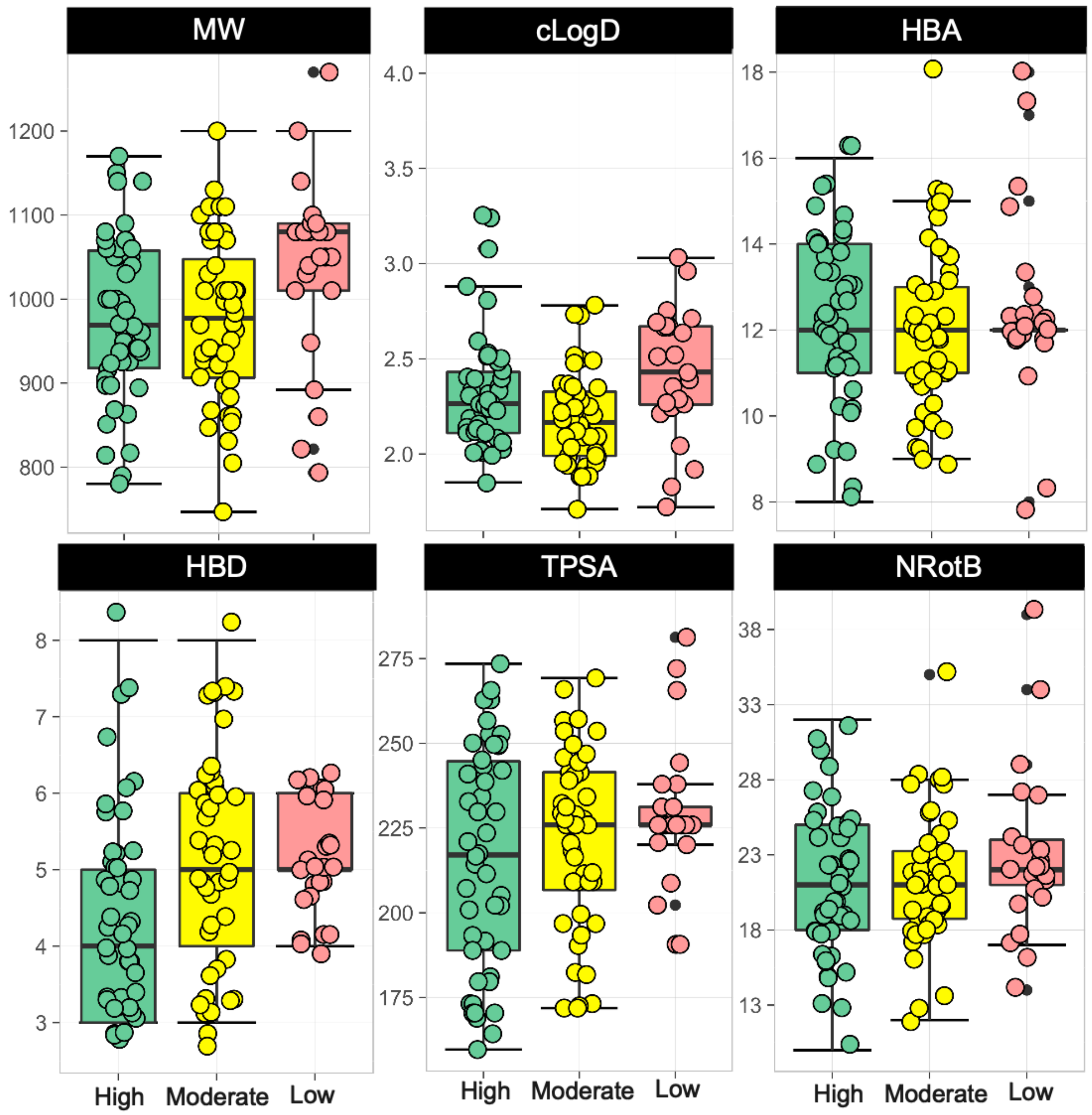

Abbreviations: Molecular weight (MW, Da), lipophilicity (cLogD), hydrogen bond acceptors and donors (HBA and HBD), topological polar surface area (TPSA, Å^2), and number of rotatable bonds (NRotB).

Box plots show the 50th percentiles as horizontal bars, the 25th and 75th percentiles as boxes, the 25th percentile minus 1.5 x the interquartile range and the 75th percentile plus 1.5 x the interquartile range as whiskers for the PROTACs. Outliers are shown both as black dots and as circles in the color of the appropriate descriptor.

Table S1. List of the molecular descriptors used to build models

No	Types	Descriptors
1	Size and Shape	Molecular Weight (MW)
2	Size and Shape	CharVol (characteristic volume)[a]
3	Size and Shape	Flexibility (number of rotatable bonds / number of bonds)
4	Countables	Number of Heavy Atoms (HA)
5	Countables	RingAtoms
6	Countables	Halogens
7	Countables	HeteroAtoms
8	Countables	RotBonds (NRotB)
9	Countables	AllBonds
10	Countables	RingCount
11	Countables	NumStereo
12	Countables	Fraction of sp3 Carbon Atoms (FSP3)
13	Countables	Hydrogen Bond Donors (HBD)
14	Countables	Hydrogen Bond Acceptors (HBA)
15	Chemical Functionality	$cLogD^{7.4}$
16	Chemical Functionality	Topological polar surface area (TPSA)
17	Chemical Functionality	Total non-polar surface area (TNSA)

[a] Molecular volume as defined by Abraham and McGowan, Chromatographia, 23, 243-246, 1987)

Figure S3. Comparison of molecular descriptors for PROTAC sets

A) Literature (Weng et al[1] combined with Maple et al.[2]) B) Original PROTAC training set (VHL + CRBN, this work)

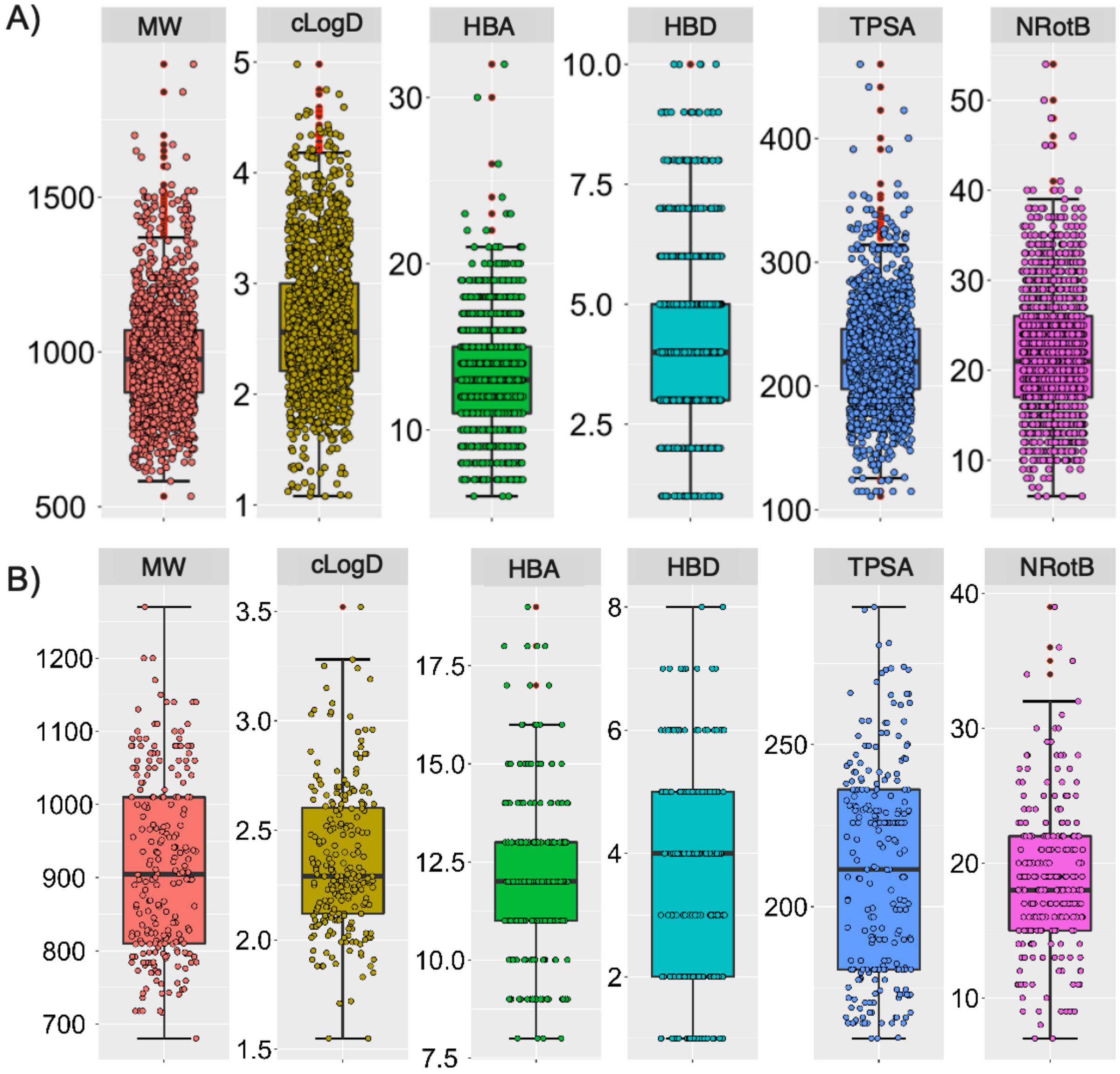

Abbreviations: Molecular weight (MW, Da), lipophilicity (cLogD), hydrogen bond acceptors and donors (HBA and HBD), topological polar surface area (TPSA, Å^2), and number of rotatable bonds (NRotB).

Box plots show the 50th percentiles as horizontal bars, the 25th and 75th percentiles as boxes, the 25th percentile minus 1.5 x the interquartile range and the 75th percentile plus 1.5 x the interquartile range as whiskers for the PROTACs. Outliers are shown both as red dots and as circles in the color of the appropriate descriptor.

Figure S4. Overall KNIME workflow

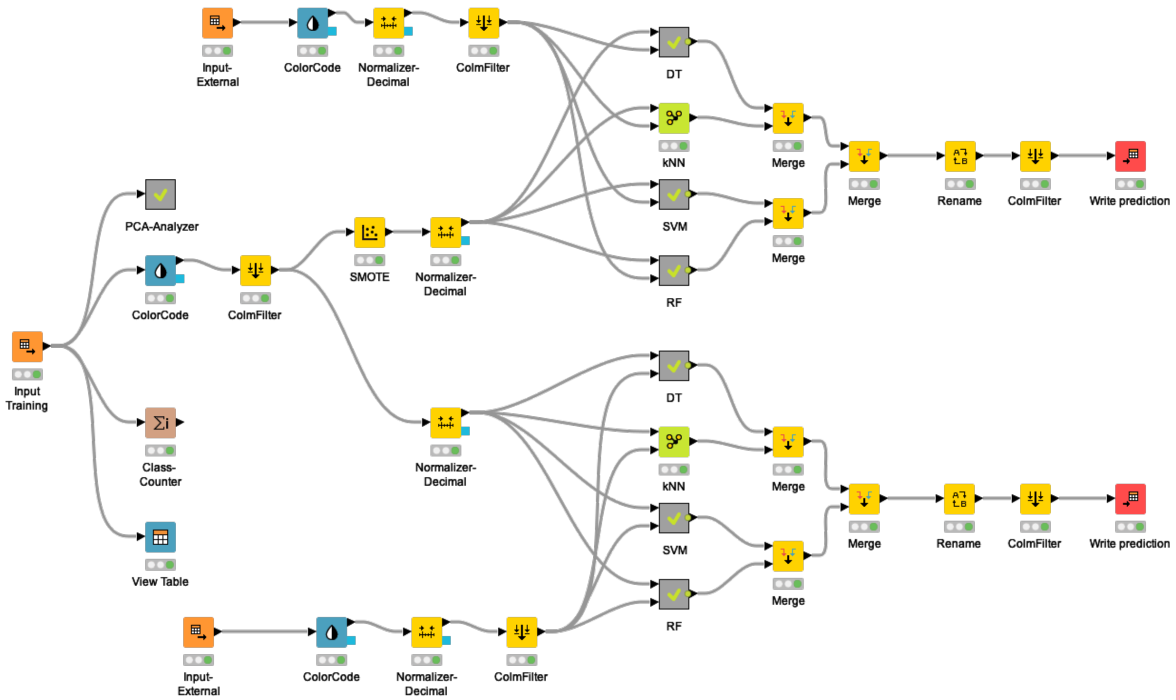

Node used for PCA and data analysis

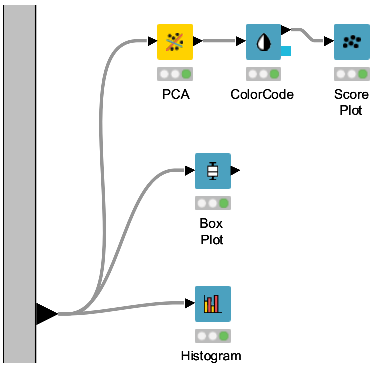

Node used for Decision Tree Model

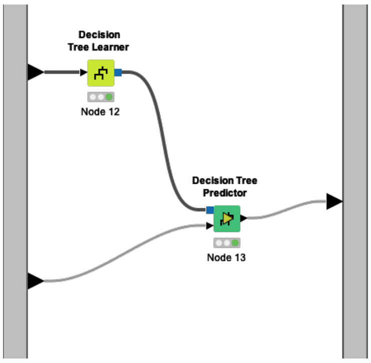

Table S2. Settings used for machine learning methods

**Decision Tree (DT)**	**Random Forest (RF)**
Quality measure: Gini Index<br>Pruning: No pruning<br>Minimum node size: 1<br>Number of threads: 8<br>Number of levels: 10	Split criterion: InfoGain<br>Number of levels: 10<br>Minimum node size: 1<br>Number of Forest: 50
**kappa Nearest Neighbour (kNN)**	**Support vector machine (SVM)**
Number of neighbours: 5	Kernel: Polynomial<br>Bias: 1.0<br>g: 2.0<br>Power: 1.0

Table S3. Summary of "CfsSubsetEval-BestFirst"-based models (VHL dataset/10-fold CV)[a]

Scenario	Method	# Descr.	Confusion Matrix				Sens.	Spec.	BCR	Acc.	κ
			TP	FN	TN	FP					
**Scenario 1** (high vs. low)	DT	4	40	6	17	8	0.87	0.68	0.77	0.80	0.56
	kNN	4	39	7	14	11	0.85	0.56	0.70	0.75	0.42
	RF	4	42	4	17	7	0.91	0.71	0.81	0.84	0.64
**Scenario 2** (high/moderate vs. low)	DT	5	84	6	7	18	0.93	0.28	0.61	0.79	0.26
	kNN	5	83	7	10	15	0.92	0.40	0.66	0.81	0.36
	RF	5	81	9	15	10	0.90	0.60	0.75	0.83	0.51
**Scenario 3** (high vs. moderate/low)	DT	1	39	30	30	16	0.57	0.65	0.61	0.60	0.21
	kNN	1	44	25	27	19	0.64	0.59	0.61	0.62	0.22
	RF	1	44	25	27	19	0.64	0.59	0.61	0.62	0.22

[a]**Abbreviations:** CV, cross-validation; #Descr., Number of descriptors obtained from the BestFirst search; TP, true positive; FN, false negative; TN, true negative; FP, false positive; Sens., sensitivity; Spec., specificity; BCR, balanced classification rate; Acc., accuracy; κ, Cohen's kappa coefficient; DT, decision tree; kNN, kappa nearest neighbour; RF, random forest.

Table S4. Compounds used for model training and cross-validation by permeability class scenario

Models	Permeability class [a]	Permeability class scenarios		
		H vs. L Scenario 1	H/M vs. L Scenario 2	H vs. M/L Scenario 3
VHL	H or H/M	46	90	46
	L or M/L	25	25	69
	Sum	71	115	115
CRBN	H or H/M	59	104	59
	L or M/L	9	9	54
	Sum	68	113	113

[a] High, moderate and low cell permeability have been denoted as H, M and L, respectively.

Figure S5. Summary of Cohan's Kappa statistics for internal validation of the global classification models constructed by combination of the VHL and CRBN training sets

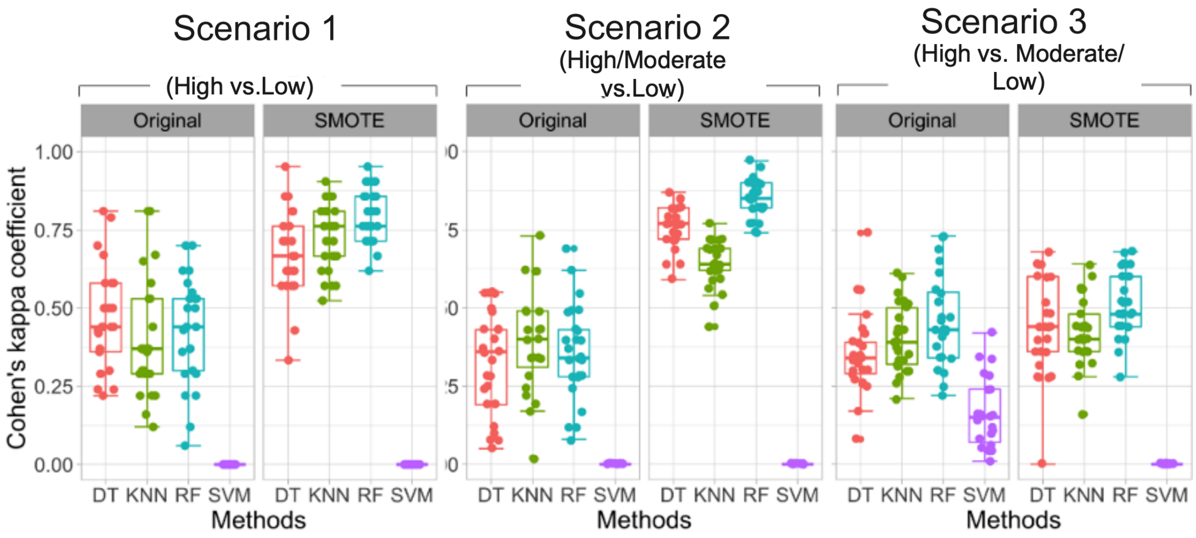

Box plots show the kappa values from 25 random seedlings, 50th percentiles as horizontal bars, the 25th and 75th percentiles as boxes, the 25th percentile minus 1.5 x the interquartile range and the 75th percentile plus 1.5 x the interquartile range as whiskers. Outliers are as circles in the color of the appropriate classifier.

Figure S6. Accuracies for CRBN (A) and VHL (B) models constructed using the original training sets.

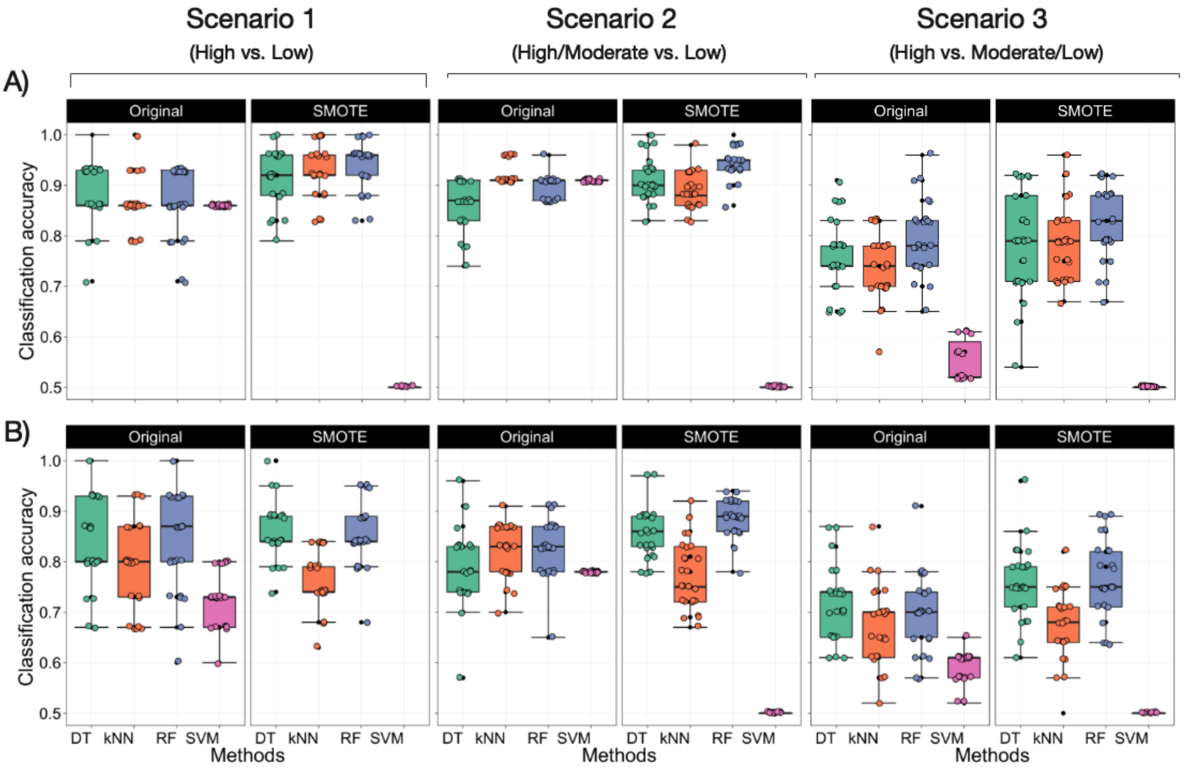

Box plots show the kappa values from 25 random seedlings, 50th percentiles as horizontal bars, the 25th and 75th percentiles as boxes, the 25th percentile minus 1.5 x the interquartile range and the 75th percentile plus 1.5 x the interquartile range as whiskers. Outliers are shown both as black dots and as circles in the color of the appropriate classifier.

Figure S7. Applicability domain assessment

Euclidean distance to mean (i.e., sum ($x^1 \dots x^n$)/n). Out of applicability domain region is highlighted with grey shade.

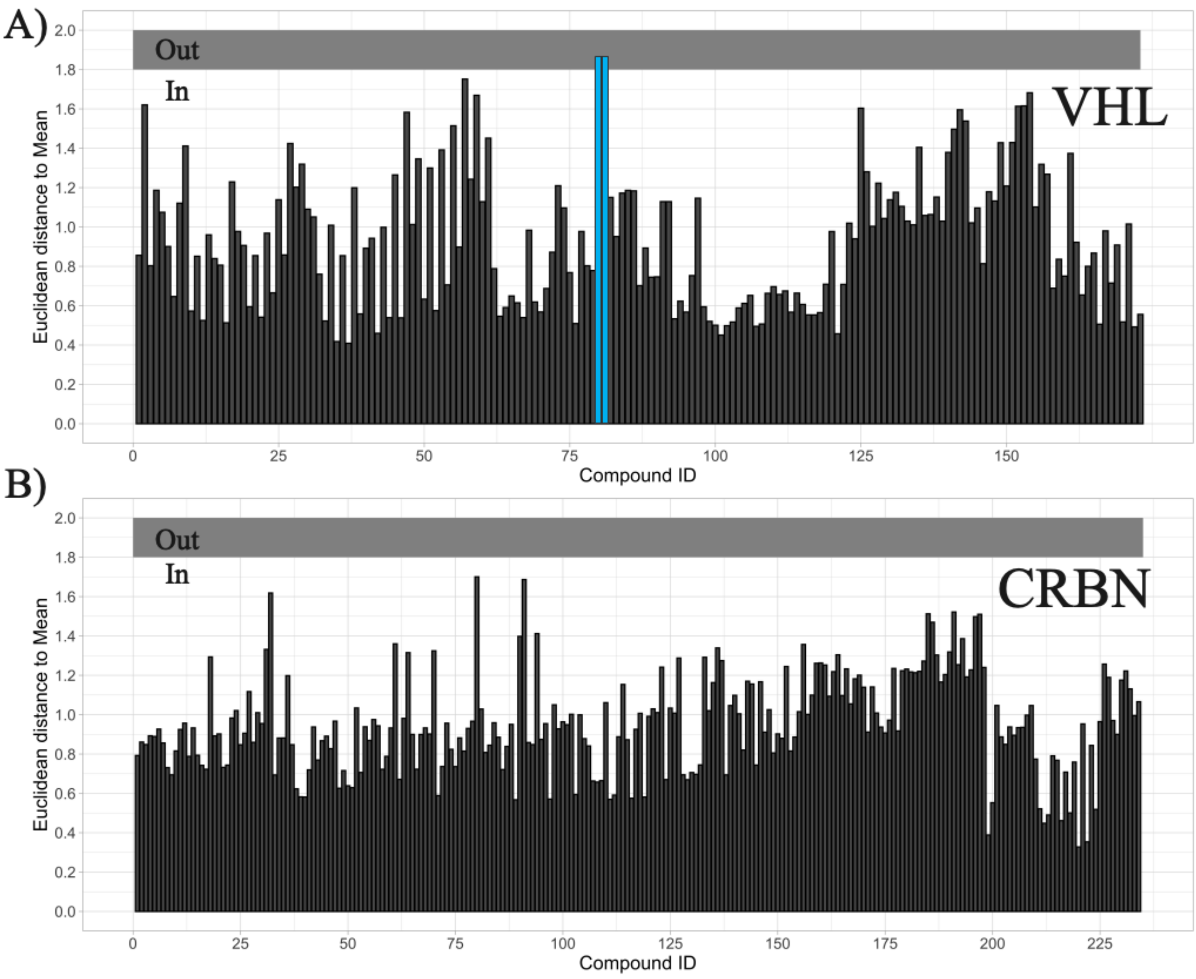

Table S5. Number of compounds by permeability class scenario for blinded test set 1[a]

Permeability class	VHL			CRBN		
	H vs. L Scenario 1	H/M vs. L Scenario 2	H vs. M/L Scenario 3	H vs. L Scenario 1	H/M vs. L Scenario 2	H vs. M/L Scenario 3
High	73	111	73	206	222	206
Low	27	27	65	14	14	30
Sum	100	138	138	220	236	234

[a] High, moderate and low cell permeability have been denoted as H, M and L, respectively.

Table S6. Summary of blinded predictions of VHL PROTACs in blinded test set 1

Scenario	Dataset	Method	Confusion Matrix				BCR	Sens.	Spec.	Acc.	κ
			TP	FN	TN	FP					
**Scenario 1** High vs. Low	Original	DT	44	29	20	7	0.67	0.6	0.74	0.64	0.27
		kNN	64	9	19	8	0.79	0.88	0.70	0.83	0.57
		RF	65	8	20	7	0.82	0.89	0.74	0.85	0.62
	SMOTE	DT	43	30	20	7	0.66	0.59	0.74	0.63	0.26
		kNN	57	16	20	7	0.76	0.78	0.74	0.77	0.47
		RF	49	24	22	5	0.74	0.67	0.81	0.71	0.40
**Scenario 2** High/Moderate vs. Low	Original	DT	60	51	21	6	0.66	0.54	0.78	0.59	0.20
		kNN	90	21	18	9	0.74	0.81	0.67	0.78	0.41
		RF	80	31	22	5	0.77	0.72	0.81	0.74	0.39
	SMOTE	DT	85	26	18	9	0.72	0.77	0.67	0.75	0.35
		kNN	70	41	19	8	0.67	0.63	0.70	0.64	0.23
		RF	75	36	22	5	0.75	0.68	0.81	0.70	0.34
**Scenario 3** High vs. Moderate/Low	Original	DT	66	7	18	47	0.59	0.9	0.28	0.56	0.17
		kNN	49	24	44	21	0.67	0.67	0.68	0.51	0.22
		RF	50	23	48	17	0.71	0.68	0.74	0.50	0.22
	SMOTE	DT	67	6	22	43	0.63	0.92	0.34	0.58	0.22
		kNN	57	16	42	23	0.71	0.78	0.65	0.55	0.26
		RF	43	30	44	21	0.63	0.59	0.68	0.46	0.16

[a] **Note:** TP, true positive; FN, false negative; TN, true negative; FP, false positive; BCR, balanced classification rate; Sens., sensitivity; Spec., specificity; Acc., accuracy; κ, Cohen's kappa coefficient; DT, decision tree; kNN, kappa nearest neighbour; RF, random forest.

Figure S8. Cohen's kappa coefficient for prediction of the permeability of the VHL and CRBN PROTACs in the blinded test set 1 using the global classification models

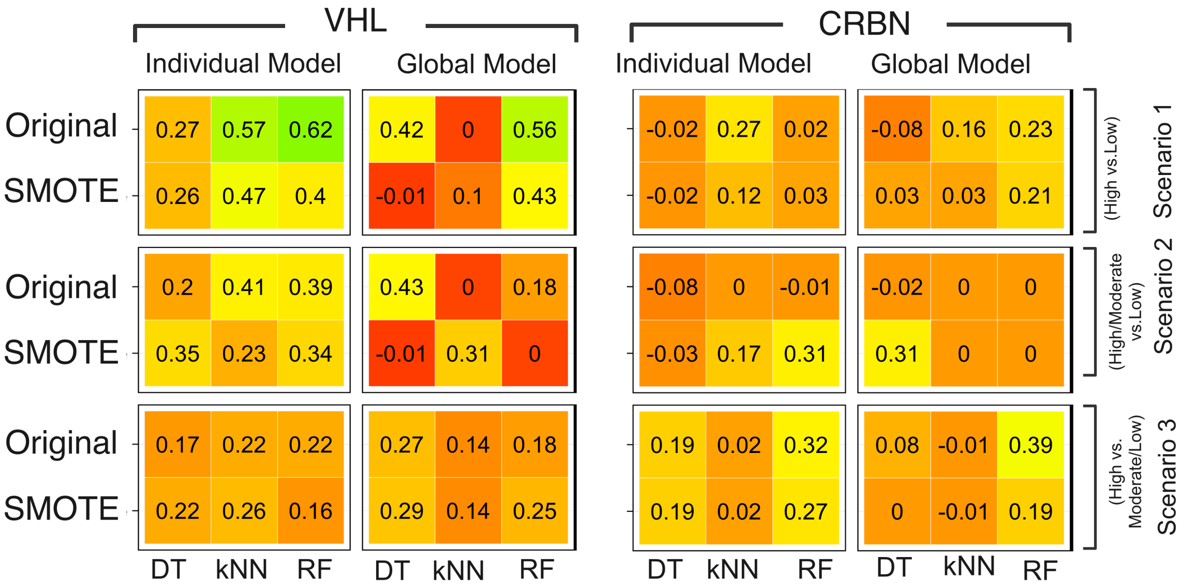

The kappa coefficient is given for the three permeability scenarios for models constructed using the DT, kNN and RF methods. Models were constructed using the individual VHL and CRBN datasets, and using the combined VHL and CRBN datasets (global model), and the corresponding SMOTE versions. Kappa coefficients have been color coded using red-orange-yellow-green for values ranging from -0.3 to 0.7.

Table S7. Summary of blinded predictions of CRBN PROTACs in blinded test set 1

Scenario	Dataset	Method	Confusion Matrix				BCR	Sens.	Spec.	Acc.	κ
			TP	FN	TN	FP					
**Scenario 1** High vs. Low	Original	DT	111	93	5	8	0.46	0.54	0.38	0.53	-0.02
		kNN	200	4	3	10	0.61	0.98	0.23	0.94	0.27
		RF	153	51	4	9	0.53	0.75	0.31	0.72	0.02
	SMOTE	DT	113	91	5	8	0.47	0.55	0.38	0.54	-0.02
		kNN	155	49	7	6	0.65	0.76	0.54	0.75	0.12
		RF	182	22	2	11	0.52	0.89	0.15	0.85	0.03
**Scenario 2** High/Moderate vs. Low	Original	DT	147	74	1	12	0.37	0.67	0.08	0.63	-0.08
		kNN	221	0	0	13	0.5	1.00	0.00	0.94	0.00
		RF	220	1	0	13	0.5	1.00	0.00	0.94	-0.01
	SMOTE	DT	217	4	0	13	0.49	0.98	0.00	0.93	-0.03
		kNN	189	32	6	7	0.66	0.86	0.46	0.83	0.17
		RF	219	2	3	10	0.61	0.99	0.23	0.95	0.31
**Scenario 3** High vs. Moderate/Low	Original	DT	177	27	13	17	0.65	0.87	0.43	0.78	0.19
		kNN	41	163	26	4	0.53	0.2	0.87	0.23	0.02
		RF	169	35	24	6	0.81	0.83	0.8	0.77	0.32
	SMOTE	DT	177	27	13	17	0.65	0.87	0.43	0.78	0.19
		kNN	39	165	26	4	0.53	0.19	0.87	0.22	0.02
		RF	170	34	20	10	0.75	0.83	0.67	0.77	0.27

[a] **Note:** TP, true positive; FN, false negative; TN, true negative; FP, false positive; BCR, balanced classification rate; Sens., sensitivity; Spec., specificity; Acc., accuracy; κ, Cohen's kappa coefficient; DT, decision tree; kNN, kappa nearest neighbour; RF, random forest.

Figure S9. Score plots of the first two principal components from PCAs of the original and retrained VHL datasets

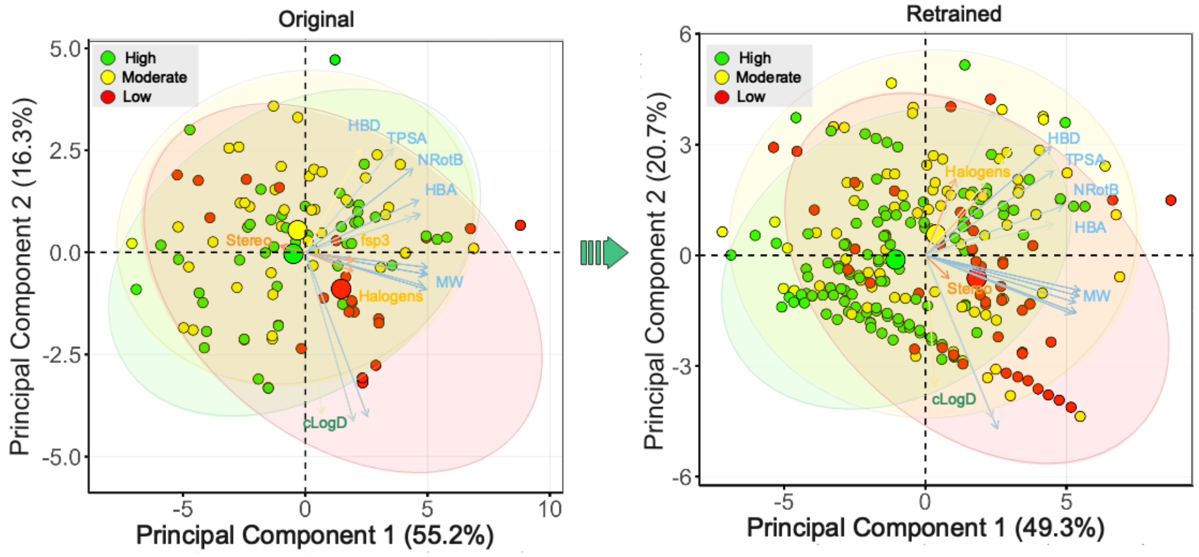

Note: The 95% confidence intervals are shown as ellipses for each permeability class.

Figure S10. Score plots of the first two principal components from PCAs of the original and retrained CRBN datasets

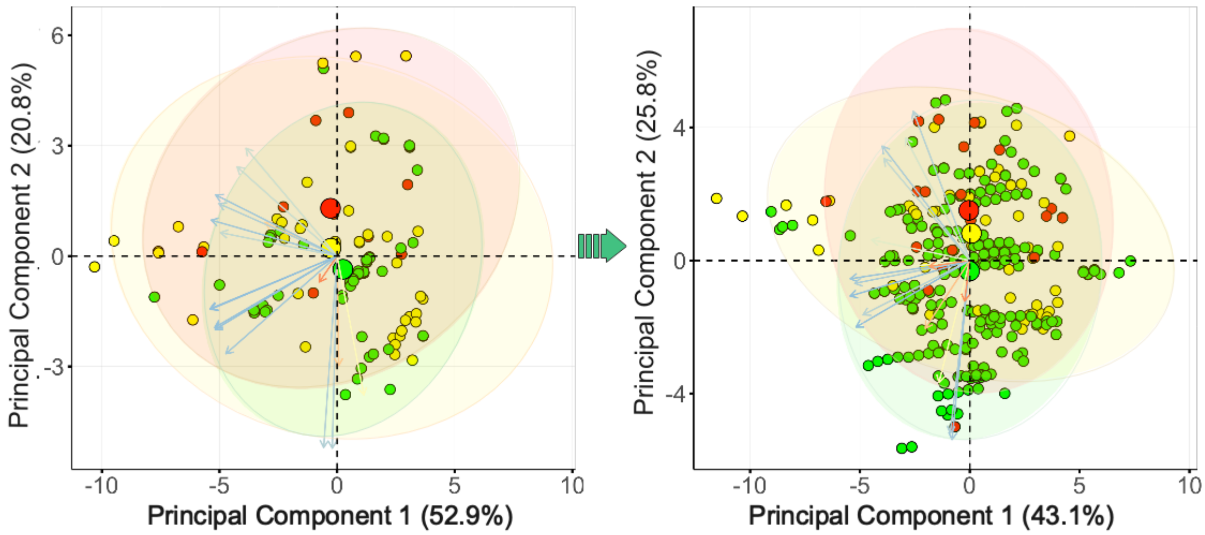

Note: The 95% confidence intervals are shown as ellipses for each permeability class.

Figure S11. Accuracies from internal test set validations of retrained CRBN (A) and VHL (B) models

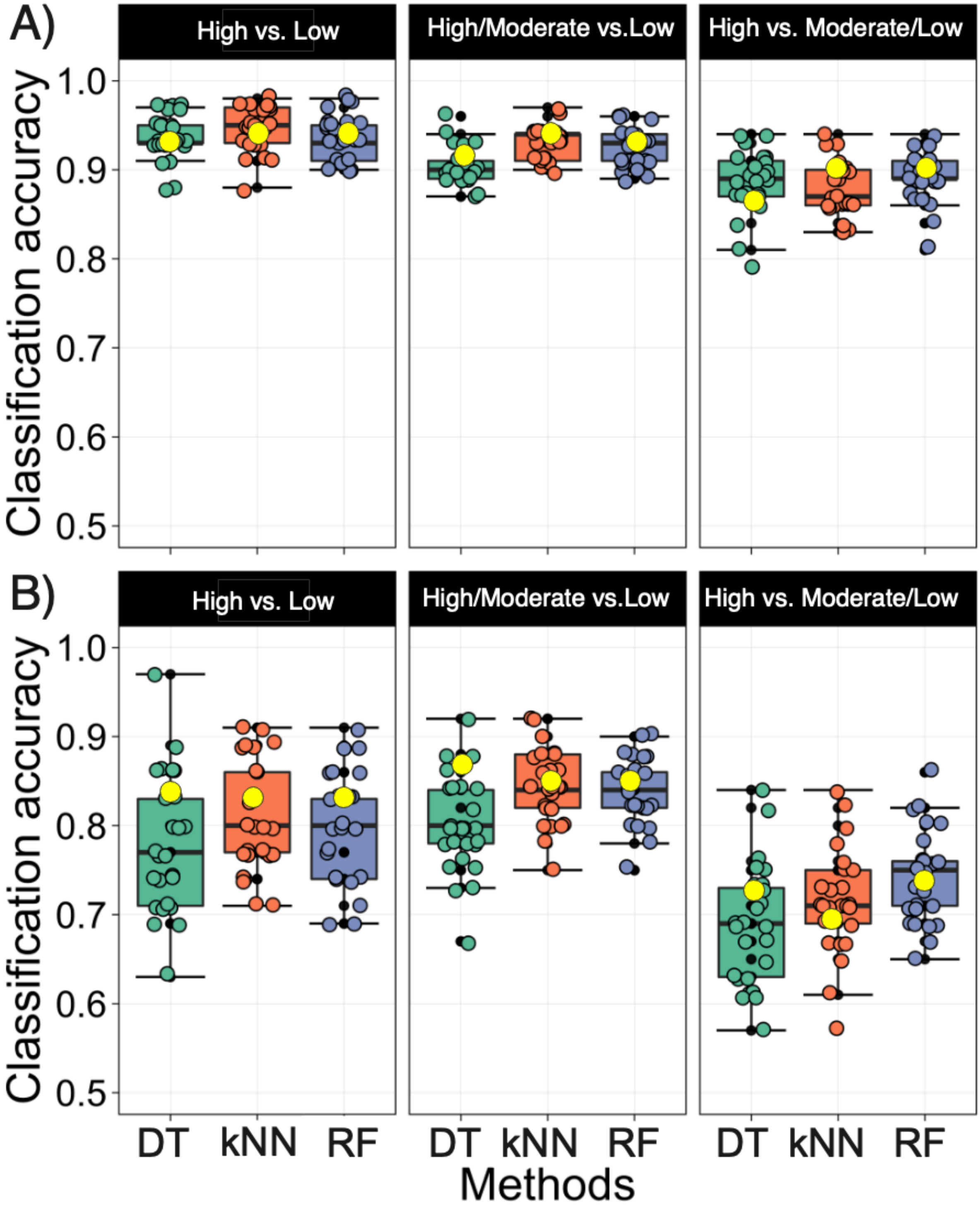

Box plots show the 50th percentiles as horizontal bars, the 25th and 75th percentiles as boxes, the 25th percentile minus 1.5 x the interquartile range and the 75th percentile plus 1.5 x the interquartile range as whiskers. Outliers are shown both as black dots and as circles in the color of the appropriate classifier.

Table S8. Summary of 10-fold cross validation of retrained BCMs.[a]

Model	Scenarios	Method	Confusion Matrix				Sens.	Spec.	BCR	Acc.	κ
			TP	FN	TN	FP					
VHL	**Scenario 1** High vs. Low	DT	107	12	36	16	0.90	0.69	0.80	0.84	0.60
		kNN	108	11	32	20	0.91	0.62	0.76	0.82	0.55
		RF	109	10	33	19	0.92	0.63	0.78	0.83	0.58
	**Scenario 2** High/Moderate vs. Low	DT	186	15	29	23	0.93	0.56	0.74	0.85	0.51
		kNN	184	17	29	23	0.92	0.56	0.74	0.84	0.49
		RF	188	13	24	28	0.94	0.46	0.70	0.84	0.44
	**Scenario 3** High vs. Moderate/Low	DT	103	31	81	38	0.77	0.68	0.72	0.73	0.45
		kNN	97	37	81	38	0.72	0.68	0.70	0.70	0.40
		RF	105	29	83	36	0.78	0.70	0.74	0.74	0.48
CRBN	**Scenario 1** High vs. Low	DT	257	8	11	12	0.97	0.48	0.72	0.93	0.49
		kNN	260	5	11	12	0.98	0.48	0.73	0.94	0.53
		RF	259	6	12	11	0.98	0.52	0.75	0.94	0.55
	**Scenario 2** High/Moderate vs. Low	DT	315	11	4	19	0.97	0.17	0.57	0.91	0.17
		kNN	320	6	8	15	0.98	0.35	0.66	0.94	0.40
		RF	319	7	5	18	0.98	0.22	0.60	0.93	0.25
	**Scenario 3** High vs. Moderate/Low	DT	250	15	60	24	0.94	0.71	0.83	0.89	0.68
		kNN	247	18	69	15	0.93	0.82	0.88	0.91	0.74
		RF	253	12	61	23	0.95	0.73	0.84	0.90	0.71

[a] **Note:** BCM, binary classification model; TP, true positive; FN, false negative; TN, true negative; FP, false positive; Sens., sensitivity; Spec., specificity; BCR, balanced classification rate; Acc., accuracy; κ, Cohen's kappa coefficient; DT, decision tree; kNN, kappa nearest neighbour; RF, random forest.

Table S9. Number of compounds by permeability class scenario for blinded test set 2[a]

Permeability class	VHL			CRBN		
	H vs. L Scenario 1	H/M vs. L Scenario 2	H vs. M/L Scenario 3	H vs. L Scenario 1	H/M vs. L Scenario 2	H vs. M/L Scenario 3
High	24	38	24	31	36	31
Low	14	14	28	4	4	9
Sum	38	52	52	35	40	40

[a] High, moderate and low cell permeability have been denoted as H, M and L, respectively.

Table S10. Performance of original models on permeability predictions of VHL blinded test set 2.[a]

Scenario	Method	Confusion Matrix				Sens.	Spec.	BCR	Acc.	κ
		TP	FN	TN	FP					
**Scenario 1** (High vs. Low)	DT	14	10	8	6	0.58	0.57	0.58	0.58	0.15
	kNN	20	4	8	6	0.83	0.57	0.70	0.74	0.42
	RF	17	7	8	6	0.71	0.57	0.64	0.66	0.28
**Scenario 2** (High/Moderate vs. Low)	DT	30	8	7	7	0.79	0.50	0.64	0.71	0.28
	kNN	31	7	2	12	0.82	0.14	0.48	0.63	-0.05
	RF	34	4	1	13	0.89	0.07	0.48	0.67	-0.04
**Scenario 3** (High vs. Moderate/Low)	DT	28	0	5	19	1.00	0.21	0.60	0.63	0.22
	kNN	21	7	8	16	0.75	0.33	0.54	0.56	0.09
	RF	19	9	14	10	0.68	0.58	0.63	0.63	0.26

[a] **Note:** TP, true positive; FN, false negative; TN, true negative; FP, false positive; Sens., sensitivity; Spec., specificity; BCR, balanced classification rate; Acc., accuracy; κ, Cohen's kappa coefficient; DT, decision tree; kNN, kappa nearest neighbour; RF, random forest.

Table S11. Performance of retrained models on permeability predictions of VHL blinded test set 2.[a]

Scenario	Method	Confusion matrix				Sens.	Spec.	BCR	Acc.	κ
		TP	FN	TN	FP					
**Scenario 1** (High vs. Low)	DT	10	14	12	2	0.42	0.86	0.64	0.58	0.23
	KNN	18	6	12	2	0.75	0.86	0.80	0.79	0.57
	RF	18	6	13	1	0.75	0.93	0.84	0.82	0.63
**Scenario 2** (High/Moderate vs. Low)	DT	28	10	11	3	0.74	0.79	0.76	0.75	0.45
	KNN	26	12	6	8	0.68	0.43	0.56	0.62	0.10
	RF	33	5	9	5	0.87	0.64	0.76	0.81	0.51
**Scenario 3** (High vs. Moderate/Low)	DT	24	4	12	12	0.86	0.50	0.68	0.69	0.37
	KNN	26	2	10	14	0.93	0.42	0.67	0.69	0.36
	RF	26	2	12	12	0.93	0.5	0.71	0.73	0.44

[a] **Note:** TP, true positive; FN, false negative; TN, true negative; FP, false positive; Sens., sensitivity; Spec., specificity; BCR, balanced classification rate; Acc., accuracy; κ, Cohen's kappa coefficient; DT, decision tree; kNN, kappa nearest neighbour; RF, random forest.

Table S12. Consensus predictions for permeability scenarios of the VHL blinded test set 2 using the retrained models

Scenario	Method [a]	Confusion Matrix [b]				BCR	Sens.	Spec.	Acc.	κ
		TP	FN	TN	FP					
**Scenario 1** High vs. Low	DT + kNN + RF	10	14	10	4	0.57	0.42	0.71	0.53	0.11
	Prediction by two	18	6	13	1	0.84	0.75	0.93	0.82	0.63
	RF + kNN	18	6	11	3	0.77	0.75	0.79	0.76	0.51
	RF+DT	10	14	12	2	0.64	0.42	0.86	0.58	0.23
	DT+kNN	10	14	10	4	0.57	0.42	0.71	0.53	0.11
	Prediction by one	18	6	14	0	0.88	0.75	1.00	0.84	0.69
	Wrong prediction	6	-	0	-	0.13	0.25	0.00	0.16	-
**Scenario 2** High/Moderate vs. Low	DT + kNN + RF	23	15	2	12	0.37	0.61	0.14	0.48	-0.24
	Prediction by two	30	8	11	3	0.79	0.79	0.79	0.79	0.52
	RF + kNN	25	13	4	10	0.47	0.66	0.29	0.56	-0.05
	RF+DT	27	11	7	7	0.61	0.71	0.50	0.65	0.19
	DT+kNN	24	14	4	10	0.46	0.63	0.29	0.54	-0.08
	Prediction by one	34	4	13	1	0.91	0.89	0.93	0.90	0.77
	Wrong prediction	4	-	1	-	0.09	0.11	0.07	0.10	-
**Scenario 3** High vs. Moderate/Low	DT + kNN + RF	7	17	23	5	0.56	0.29	0.82	0.58	0.12
	Prediction by two	12	12	25	3	0.70	0.50	0.89	0.71	0.40
	RF + kNN	7	17	23	5	0.56	0.29	0.82	0.58	0.12
	RF+DT	10	14	23	5	0.62	0.42	0.82	0.63	0.24
	DT+kNN	7	17	23	5	0.55	0.29	0.82	0.58	0.12
	Prediction by one	15	9	28	0	0.81	0.63	1.00	0.83	0.64
	Wrong prediction	9	-	0	-	0.19	0.38	0.00	0.17	-

[a] All three: RF+KNN+DT; Any of two methods agree: RF+DT or RF+kNN or DT+kNN; Any one method prediction: DT or kNN or RF.

[b] TP, true positive; FN, false negative; TN, true negative; FP, false positive.

BCR, balanced classification rate; Sens., sensitivity; Spec., specificity; Acc., accuracy; κ, Cohen's kappa coefficient.

Table S13. Performance of retrained models on permeability predictions of CRBN blinded test set 2.[a]

Scenario	Method	Confusion Matrix				BCR	Sens.	Spec.	Acc.	κ
		TP	FN	TN	FP					
**Scenario 1** (High vs. Low)	DT	31	0	0	4	0.5	1.0	0.0	0.89	0.0
	KNN	31	0	0	4	0.5	1.0	0.0	0.89	0.0
	RF	31	0	0	4	0.5	1.0	0.0	0.89	0.0
**Scenario 2** (High/Moderate vs. Low)	DT	36	0	0	4	0.5	1.0	0.0	0.9	0.0
	KNN	36	0	0	4	0.5	1.0	0.0	0.9	0.0
	RF	36	0	0	4	0.5	1.0	0.0	0.9	0.0
**Scenario 3** (High vs. Moderate/Low)	DT	28	8	1	3	0.51	0.78	0.25	0.73	0.02
	KNN	28	8	0	4	0.39	0.78	0.0	0.70	-0.2
	RF	36	0	0	4	0.5	1.0	0.0	0.90	0.0

[a] **Note:** TP, true positive; FN, false negative; TN, true negative; FP, false positive; BCR, balanced classification rate; Sens., sensitivity; Spec., specificity; Acc., accuracy; κ, Cohen's kappa coefficient; DT, decision tree; kNN, kappa nearest neighbour; RF, random forest.

Figure S12. Descriptor importance for retrained VHL and CRBN models.

A) Mean values from RF models for high vs. low, high/moderate vs. low and high vs. moderate/low scenarios

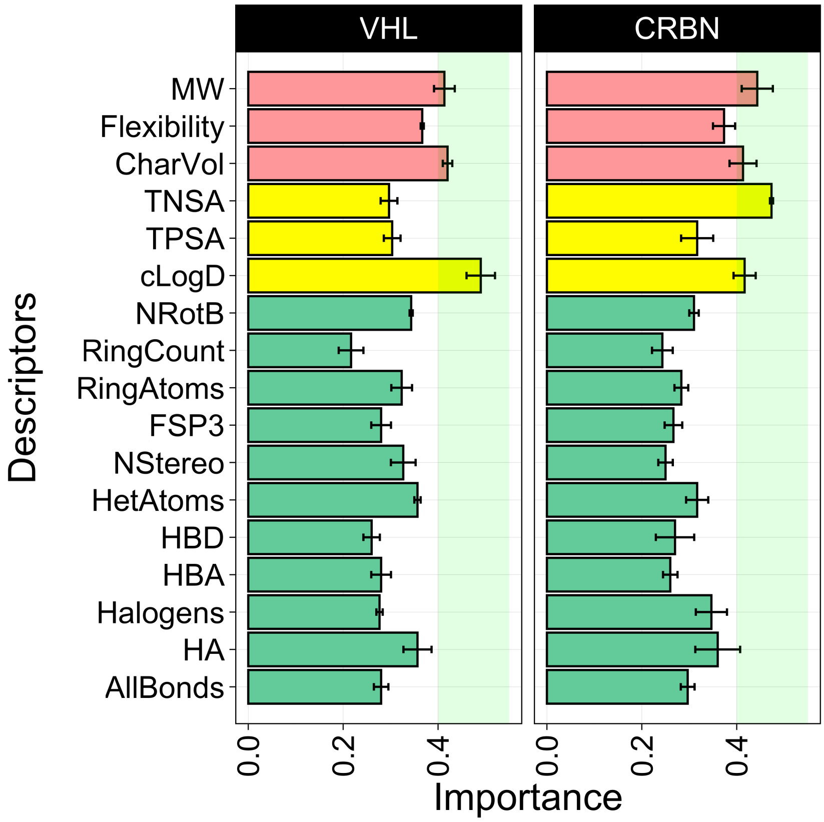

Note: Red bars: size and shape descriptors; Yellow: Chemical functionalities descriptors; Green: Countable descriptors. Descriptor importance >0.4 is highlighted with transparent green colour.

B) Values from individual RF models for high vs. low, high/moderate vs. low and high vs. moderate/low scenarios

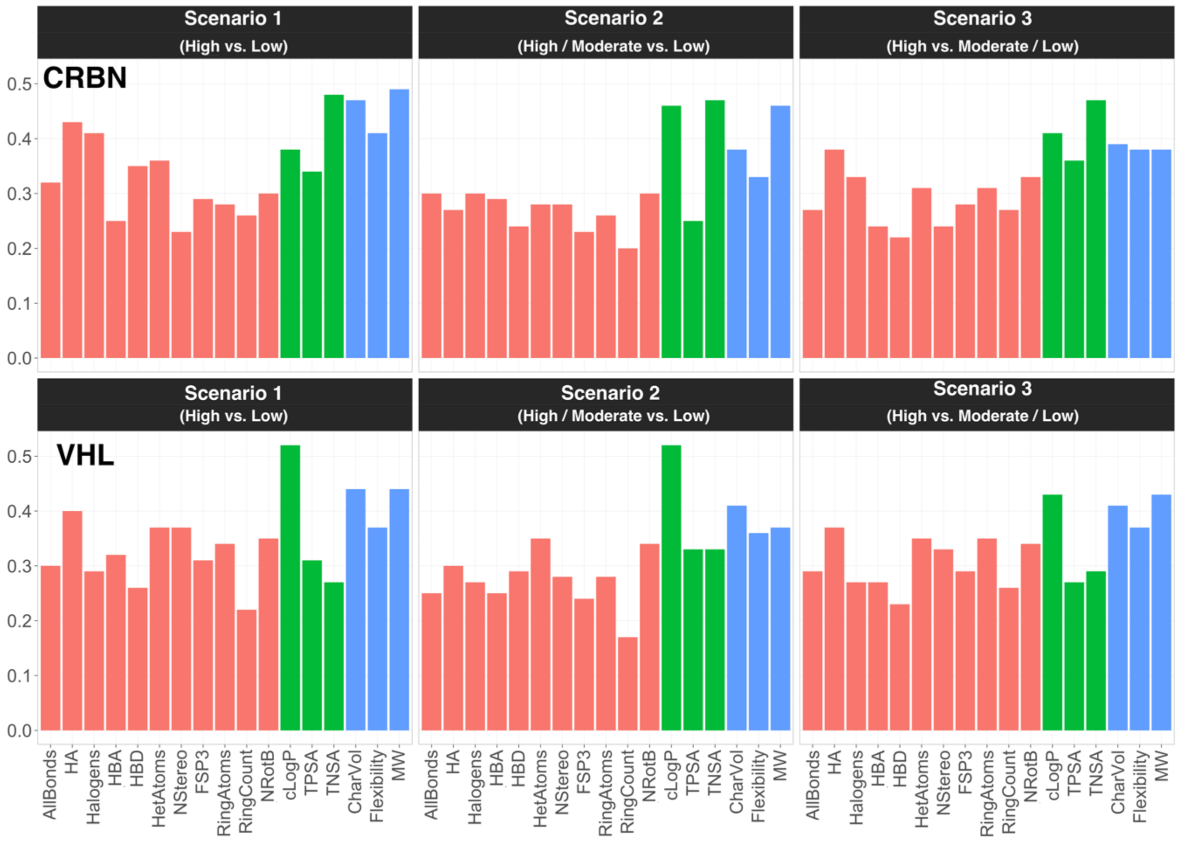

Note: Red: Countable descriptors; Green: Chemical functionalities descriptors; Blue: size and shape descriptors.

Figure S13. Distribution of the molecular descriptors of Lipinski's and Veber's guidelines for the POI ligand part (n=46) of the VHL PROTACs in the training set and blinded test set 1 (n=253)

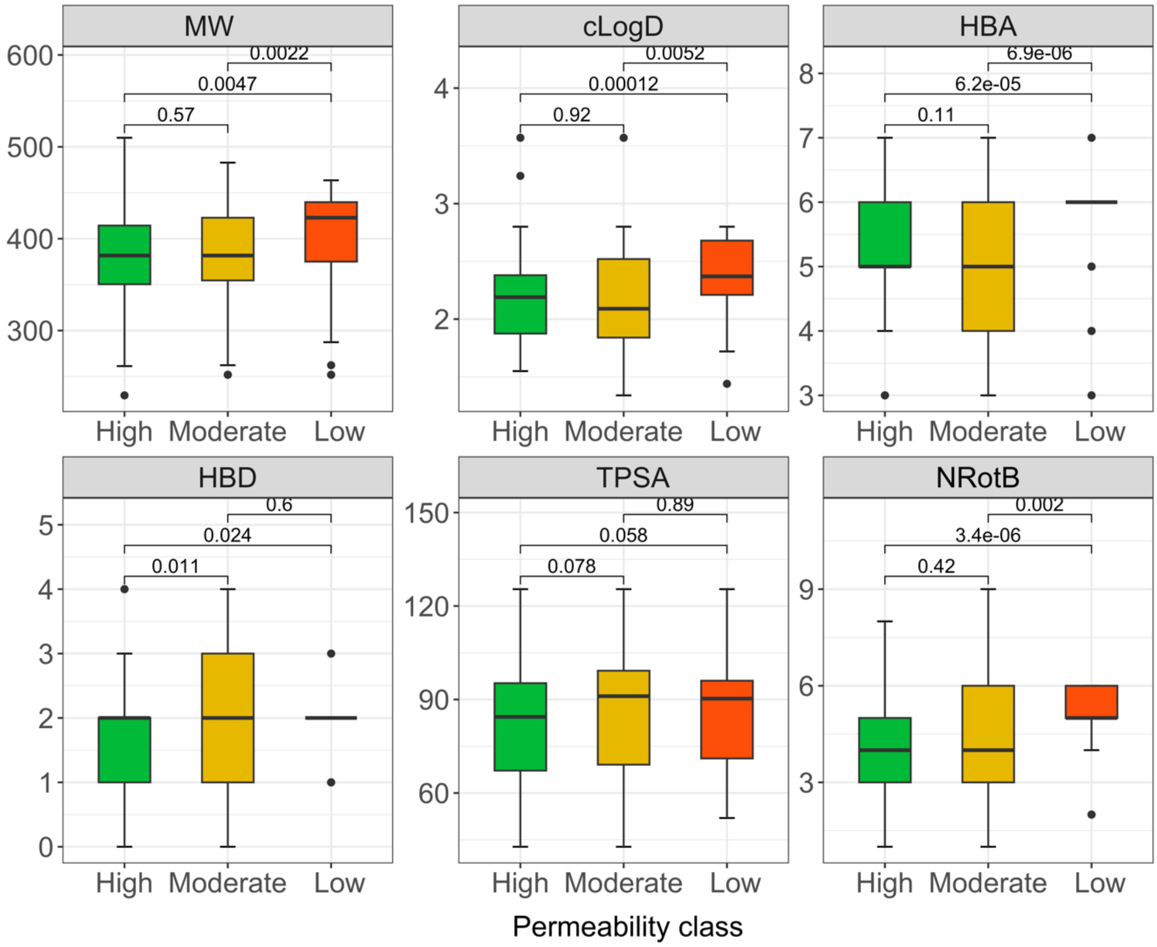

Distributions have been calculated for the POI ligands of the PROTACS in each of the three permeability classes. Box plots show the 50th percentiles as horizontal bars, the 25th and 75th percentiles as boxes, the 25th percentile minus 1.5 x the interquartile range and the 75th percentile plus 1.5 x the interquartile range as whiskers. Outliers are shown both as black dots. Statistical analysis was performed using Wilcoxon´s non-parametric test.

Figure S14. Molecular descriptor distribution for the three CRBN datasets

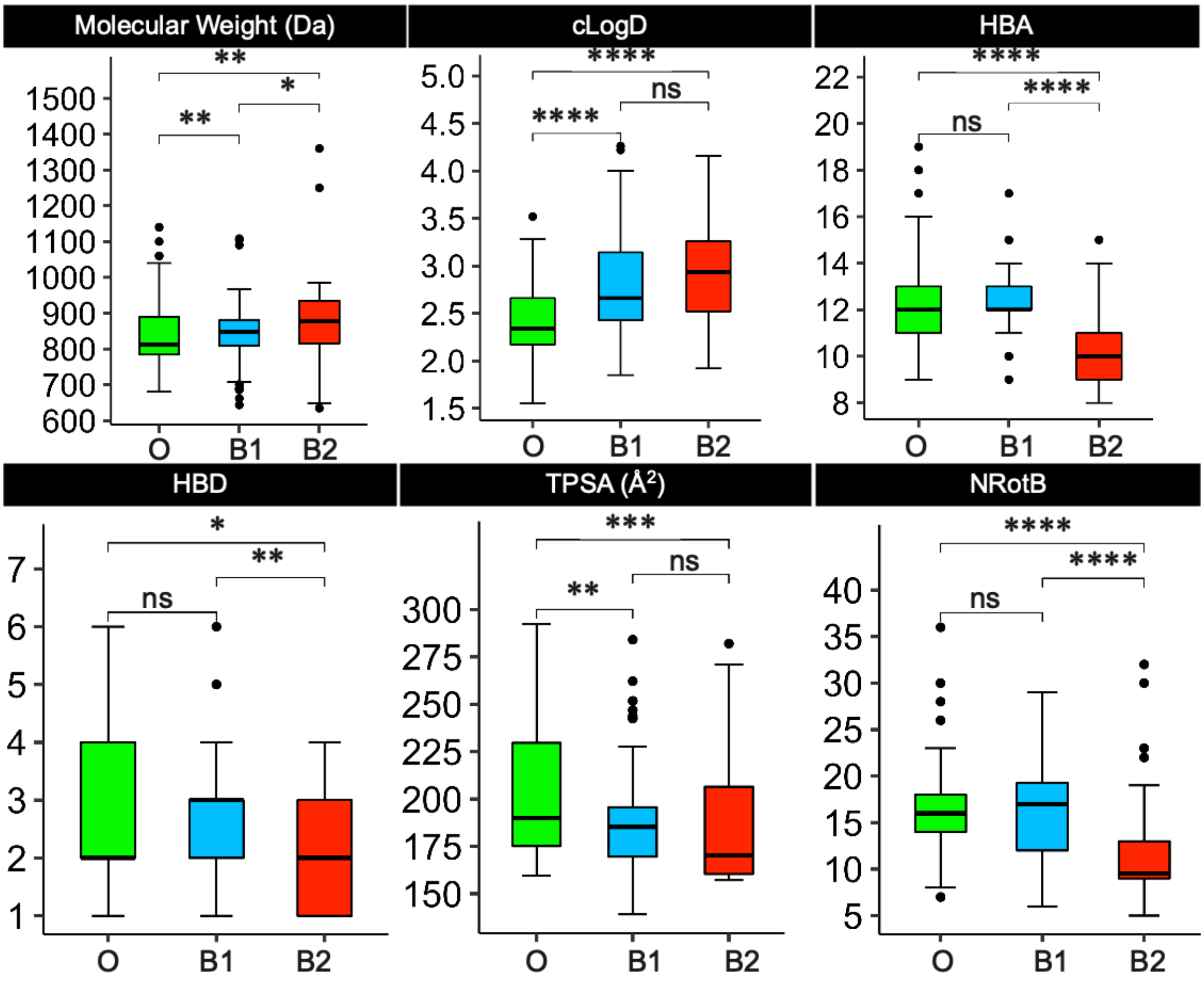

Note: O: original dataset, B1: blinded test set 1, B2: blinded test set 2
Wilcoxon test p-values: * ≤ 0.05, ** ≤ 0.01, *** ≤ 0.001, **** ≤ 0.0001.

Figure S15. Molecular descriptor distribution for the three VHL datasets

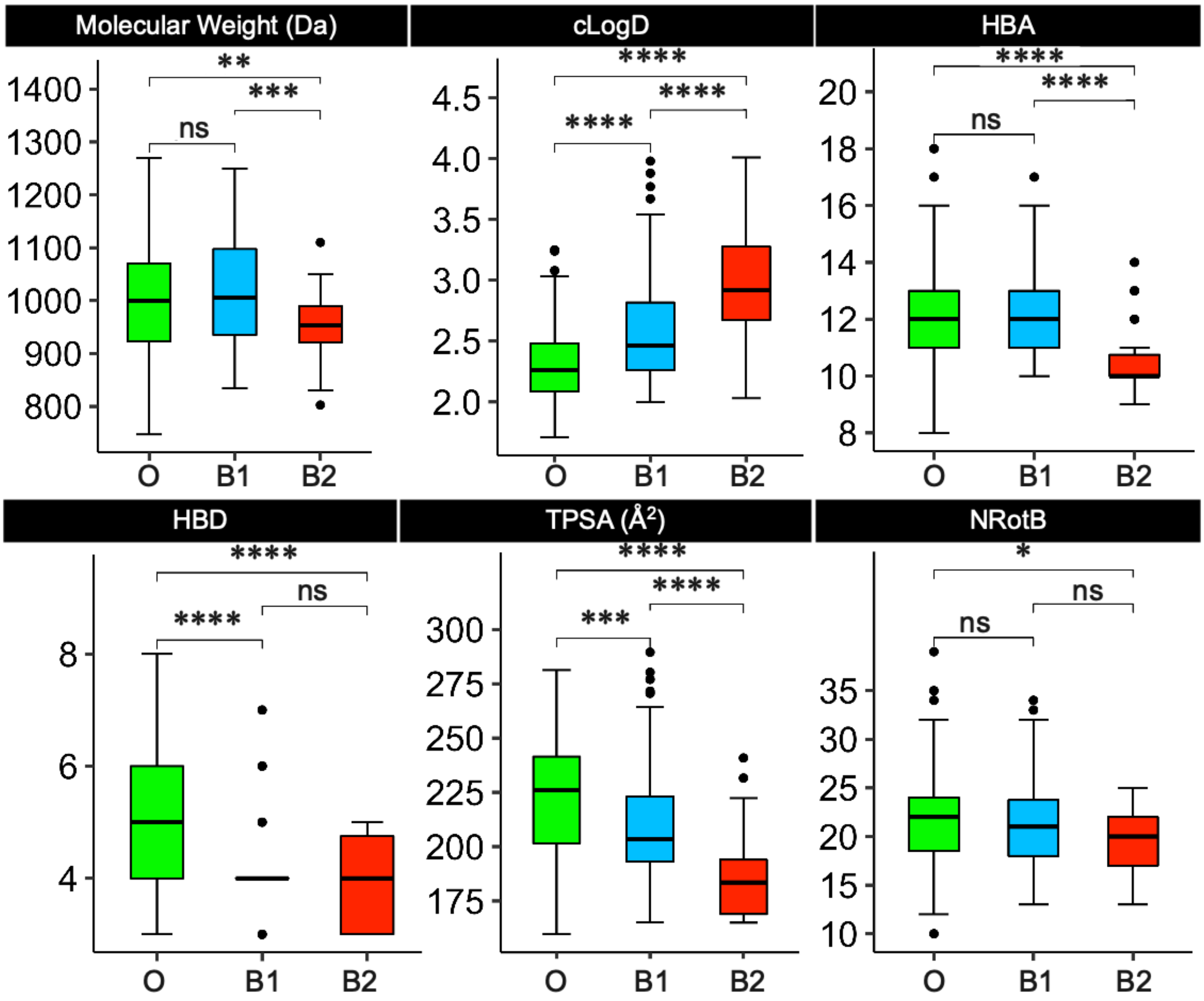

Note: O: original dataset, B1: blinded test set 1, B2: blinded test set 2
Wilcoxon test p-values: * ≤ 0.05, ** ≤ 0.01, *** ≤ 0.001, **** ≤ 0.0001.

Table S14. Distribution of all 17 molecular descriptors for the three VHL datasets

Descriptors	Original	Blind1	Blind2	Property change
MW (Da)	1000	1005	953	Slightly reduced
CharVol	751	766	721	Slightly reduced
Flex	0.27	0.26	0.26	No significant change
HA	72	73	69	Slightly reduced
RingAtoms	38	41	41	Slightly increased
Halogens	1	0	2	Slightly increased
HeteroAtoms	20	18	19	No significant change
RotBonds	22	21	20	Slightly reduced
AllBonds	78	79	76	No significant change
RingCount	7	8	8	Slightly increased
NumStereo	3	3	3	No significant change
FSP3	0.45	0.46	0.46	No significant change
HBD	5	4	4	Slightly reduced
HBA	12	12	10	Slightly reduced
TPSA ($Å^2$)	226	204	184	**Significantly reduced**
TNSA ($Å^2$)	810	840	818	No significant change
$cLogD^{7.4}$	2.3	2.5	2.9	**Significantly increased**

Note: The tabulated data are median values

Figure S16. Distribution of the PROTACs by permeability class for the three VHL datasets

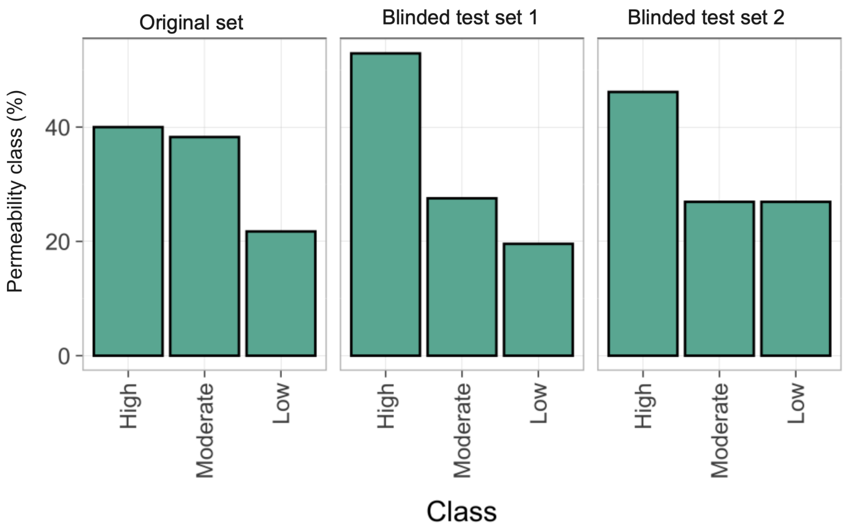

References

[1] G. Weng, C. Shen, D. Cao, J. Gao, X. Dong, Q. He, B. Yang, D. Li, J. Wu, T. Hou, *Nucleic Acids Res* **2021**, *49*, D1381-D1387.

[2] H. J. Maple, N. Clayden, A. Baron, C. Stacey, R. Felix, *Medchemcomm* **2019**, *10*, 1755-1764.
